# Synthetic viability by BRCA2 and PARP1/ARTD1 deficiencies

**DOI:** 10.1038/ncomms12425

**Published:** 2016-08-08

**Authors:** Xia Ding, Arnab Ray Chaudhuri, Elsa Callen, Yan Pang, Kajal Biswas, Kimberly D. Klarmann, Betty K. Martin, Sandra Burkett, Linda Cleveland, Stacey Stauffer, Teresa Sullivan, Aashish Dewan, Hanna Marks, Anthony T. Tubbs, Nancy Wong, Eugen Buehler, Keiko Akagi, Scott E. Martin, Jonathan R. Keller, André Nussenzweig, Shyam K. Sharan

**Affiliations:** 1 Mouse Cancer Genetics Program, National Cancer Institute, NIH, Frederick, MD 21702, USA; 2 Laboratory of Genome Integrity, National Cancer Institute, NIH, Bethesda, MD 20893, USA; 3 Leidos Biomedical Research, Inc., Frederick National Laboratory for Cancer Research, Frederick, MD 21702, USA; 4 Chemical Genomics Center, National Center for Advancing Translational Sciences, NIH, Rockville, MD 20850, USA; 5 Human Cancer Genetics Program, The Ohio State University Comprehensive Cancer Center, Columbus, OH 43210, USA; 6 Present address: Department of Discovery Oncology, Genentech, Inc., South San Francisco, CA 94080, USA

## Abstract

Poly (ADP-ribose) polymerase (PARP) inhibitor (PARPi) olaparib has been approved for treatment of advanced ovarian cancer associated with *BRCA1* and *BRCA2* mutations. *BRCA1*- and *BRCA2*-mutated cells, which are homologous recombination (HR) deficient, are hypersensitive to PARPi through the mechanism of synthetic lethality. Here we examine the effect of PARPi on HR-proficient cells. Olaparib pretreatment, PARP1 knockdown or *Parp1* heterozygosity of *Brca2*^*cko/ko*^ mouse embryonic stem cells (mESCs), carrying a null (*ko*) and a conditional (*cko*) allele of *Brca2*, results in viable *Brca2*^*ko/ko*^ cells. PARP1 deficiency does not restore HR in *Brca2*^*ko/ko*^ cells, but protects stalled replication forks from MRE11-mediated degradation through its impaired recruitment. The functional consequence of *Parp1* heterozygosity on BRCA2 loss is demonstrated by a significant increase in tumorigenesis in *Brca2*^*cko/cko*^ mice. Thus, while olaparib efficiently kills BRCA2-deficient cells, we demonstrate that it can also contribute to the synthetic viability if PARP is inhibited before BRCA2 loss.

## Abstract (web summary)

The PARP inhibitor olaparib is an approved treatment method for women with BRCA1 and BRCA2 mutation associated cancers. Here the authors show that olaparib can contribute to synthetic viability of cells if PARP1 is inhibited before BRCA2 loss.

## Graphical abstract

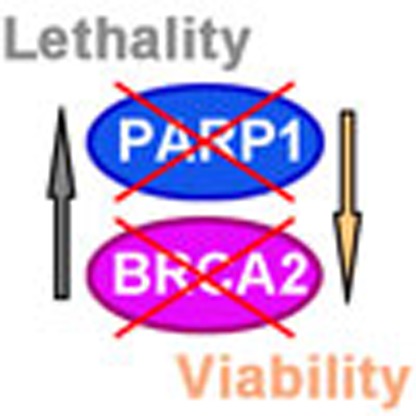

Women with a deleterious mutation in *BRCA1* or *BRCA2* have up to 70% risk of developing breast cancer by the age of 70 (ref. 1). It is well established that BRCA1 and BRCA2 function as tumour suppressors by maintaining genomic integrity. Both proteins are required for the repair of double-strand breaks (DSBs) by homologous recombination (HR) and also for the stability of stalled replication forks^1^,^2^,^3^. Their role in HR has been utilized to develop a therapeutic strategy that is based on the synthetic lethality of BRCA-deficient tumour by poly (ADP-ribose) polymerase (PARP or ADP-ribosyltransferase diphtheria toxin-like, ARTD) inhibitors^4^,^5^,^6^,^7^.

PARPs consist of a family of enzymes that catalyse the formation of ADP-ribose polymers from NAD^+^ to glutamate, aspartate or lysine residues of target proteins. At least 18 members of the PARP family have been identified based on the presence of a conserved catalytic domain. Poly ADP-ribosylation or parylation is a dynamic process as the ADP-ribose polymers can be rapidly degraded by poly (ADP-ribose) glycohydrolase and poly (ADP-ribose) hydrolase 3 (refs 8, 9). PARP1, the founding member of the PARP family, has been shown to be stimulated in response to DNA damage. Parylation of target proteins by PARP1 results in decondensation of the chromatin near the site of DNA break, which is thought to facilitate the recruitment of DNA repair proteins^10^. Nevertheless, loss of PARP1 results in viable mice with no apparent defect except for the development of spontaneous tumours after a long latency and mild sensitivity to γ-radiation and alkylating agents^11^.

PARP inhibitors effectively kill *BRCA*-deficient tumour cells. Originally, it was proposed that PARP inhibitors caused an increase of single-strand breaks, which are converted to DSBs during replication. Replication-associated DSBs are irreparable in the absence of HR, and are therefore toxic in the setting of BRCA deficiency^4^,^5^. Other plausible explanations for synthetic lethality include trapping of PARP1 at sites of DSBs or an increase in toxic non-homologous end joining in PARP1-deficient cells^12^,^13^. Regardless of the precise mechanism, PARP inhibitors such as olaparib continue to be the most promising and attractive treatment option for BRCA-deficient tumours because of their selectivity and apparent lack of toxicity in normal cells. Moreover, the repertoire of tumours that can be treated with PARP inhibitor (PARPi) is expanding to tumours with mutation in other genes associated with HR^14^.

Although only mild side effects have been reported from PARPi treatment^6^,^15^,^16^, its off-target effects are poorly understood^8^. In this study, rather than treating HR-deficient cells with a PARPi, we treated HR-proficient *Brca2*^*cko/ko*^ mouse embryonic stem cells (mESCs) to examine its effect on normal cells. We primarily used mESCs because they predominantly use HR to repair damaged DNA, and also loss of PARP1 does not affect their survival^17^,^18^. Surprisingly, we found that chemical inhibition, as well as PARP1 knockdown and heterozygosity of *Parp1* rescued the lethality of *Brca2*-null mESC without restoring HR. PARP1 deficiency suppressed the recruitment of MRE11 nuclease to stalled replication fork, which contributed to fork protection in cells lacking BRCA2.

## Results

### Rescue of *Brca2* ^*ko/ko*^ mESC by PARP inhibition or deficiency

PARPi are selectively toxic to HR-deficient cells^4^,^5^. However, effects of PARPi are not limited to HR-deficient cells, as treatment of HR-proficient cells with different doses of olaparib resulted in suppression of CHK1 activation in response to replicative stress (Supplementary Fig. 1a), as reported previously^19^. To further examine the effect of olaparib on HR-proficient cells, we used PL2F7 mESCs that have one functionally null allele of *Brca2* and the other is a conditional allele (*Brca2*^*cko/ko*^) (Supplementary Fig. 1b)^20^. We treated these cells with different doses of olaparib for 48–72 h and then deleted the *cko* allele by transient expression of CRE. Cell cycle analysis showed these olaparib regimens did not overall significantly affect the cell cycle distribution (Supplementary Fig. 1c,d) or TRP53 and p19ARF stress responses (Supplementary Fig. 1h).

**Figure 1:**
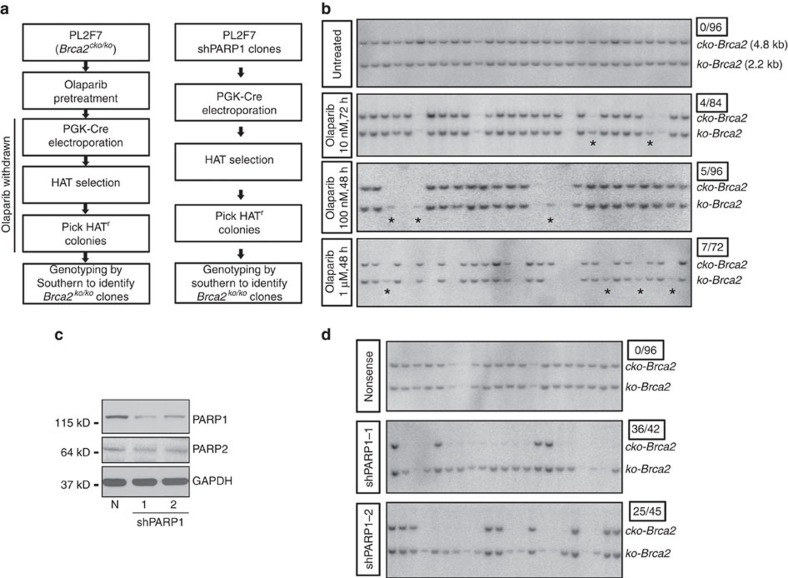
PARP inhibition or PARP1 deficiency rescues lethality of *Brca2*^*ko/ko*^ mESC. (**a**) Workflow to test the rescue of *Brca2*^*ko/ko*^ mESC lethality. (**b**) Representative Southern blot showing rescue of *Brca2*^*ko/ko*^ mESC by olaparib pretreatment in PL2F7 cells. Asterisks indicate the rescued clones. Ratio of the number of rescued clones and total numbers of HAT^r^ clones analysed are shown in the box on the right corner (same as below). (**c**) Western blot showing PARP1 level in stable knockdown clones. N, nonsense. (**d**) Representative Southern blot showing rescue of *Brca2*^*ko/ko*^ mESC by PARP1 knockdown.

After expression of CRE, we selected the recombinant clones in HAT (hypoxanthine, aminopterin and thymidine) media because CRE-mediated deletion of *cko* generates a functional *HPRT* minigene (Fig. 1a). Genotyping of the colonies did not reveal any *Brca2*^*ko/ko*^ clones in untreated cells (*n*=96), consistent with the fact that BRCA2 is essential for viability^21^. Remarkably, olaparib pretreatment resulted in a number of viable *Brca2*^*ko/ko*^ cells at all doses tested (Fig. 1b) ranging from 4 to 8% of clones.

To test whether PARP1 deficiency in *Brca2*^*ko/ko*^ cells would have similar functional consequences as observed with chemical inhibition of PARP, we generated two stably knocked-down clones using two different shRNAs against *Parp1* (Fig. 1c). PARP1 stable knockdown clone had similar cell cycle distribution compared with nonsense control clone (Supplementary Fig. 1e–g). We again obtained several HAT-resistant mESC clones after *cko* deletion. Genotyping of the clones revealed that up to 86% were *Brca2*^*ko/ko*^ (Fig. 1d). These results demonstrate that PARP1 deficiency rescues the viability of *Brca2*^*ko/ko*^ mESC.

To further strengthen these findings, we generated *Parp1* knockout clones in PL2F7 cells by using CRISPR-Cas9 system to target exon 2 (Supplementary Fig. 2a,b). We used one *Parp1* heterozygous (*Parp1*^*ko/+*^) clone and a compound heterozygous clone that was functionally null (*Parp1*^*ko/ko*^) for the mESC rescue experiment (Supplementary Fig. 2c–f). We obtained several *Brca2*^*ko/ko*^ mESC clones from *Parp1*^*ko/+*^ PL2F7 cells (61%) confirming that the rescue of BRCA2 loss-induced mESC lethality by PARP1 deficiency (Supplementary Fig. 2g). However, no *Brca2*^*ko/ko*^ mESC was obtained when we used *Parp1*^*ko/ko*^ PL2F7 cells suggesting that these cells are sensitive to complete loss of both PARP1 and BRCA2, and residual PARP1 activity is required for survival of *Brca2*^*ko/ko*^ mESC. Furthermore, the percentage of *Brca2*^*ko/ko*^ mESC obtained on a *Parp1* heterozygous background or by stable knockdown of PARP1 is high compared with 4–8% observed when cells were transiently treated with olaparib. This suggests that either prolonged PARP1 deficiency supports viability of *Brca2*^*ko/ko*^ mESC better than transient inhibition or that PARPi, in addition to rescuing the BRCA2 loss, also results in toxicity to the cells.

We further validated the genetic interaction between BRCA2 and PARP1 by examining the consequence of PARP1 deficiency on the phenotype of *Brca2*^*ko/ko*^ embryos. While no viable *Brca2*^*ko/ko*^ pups were obtained from *Parp1*^*ko/+*^*;Brca2*^*ko/+*^ intercross, we did observe a significant rescue of the *Brca2*^*ko/ko*^ embryos on *Parp1*^*ko/+*^ genetic background (Supplementary Fig. 3c,d). At E8.5, *Parp1*^*+/+*^*;Brca2*^*ko/ko*^ embryos were severely retarded and disorganized, whereas *Parp1*^*ko/+*^*;Brca2*^*ko/ko*^ embryos had undergone gastrulation and exhibited well-developed extra embryonic structures (Supplementary Fig. 3c). At E10.5, we did not obtain any *Parp1*^*ko/+*^*;Brca2*^*ko/ko*^ embryos (Supplementary Fig. 3e) indicating that these embryos die between E9.5 and E10.5. Consistent with the mESC results, we did not obtain any *Parp1*^*ko/ko*^*;Brca2*^*ko/ko*^ embryos at E8.5 by either *Parp1*^*ko/+*^*;Brca2*^*ko/+*^ intercross or *Parp1*^*ko/+*^*;Brca2*^*ko/+*^ crossed with *Parp1*^*ko/ko*^*;Brca2*^*ko/+*^ (Supplementary Fig. 3a,b), indicating that complete loss of PARP1 and BRCA2 is detrimental to mESC, as well as mouse embryos. These results suggest that PARP1 is essential for the viability of *Brca2* null cells, likely due to its wide-ranging biological functions^8^.

### HR is not restored by PARP1 deficiency in *Brca2* ^*ko/ko*^ mESC

**Figure 2:**
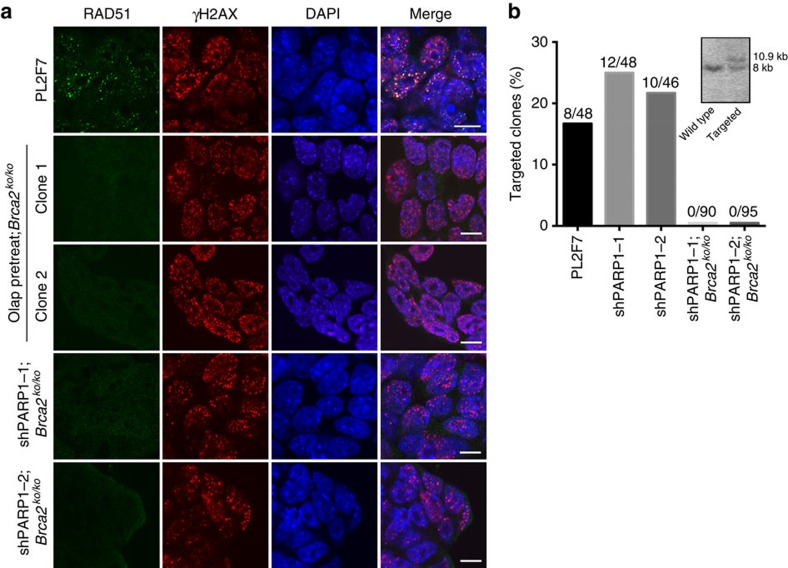
HR is not restored by PARP1 deficiency in *Brca2*^*ko/ko*^ mESC. (**a**) Immunofluorescence images showing RAD51 foci formation 5 h after 10 Gy irradiation (IR). RAD51 foci were assessed at a single time point with a single dose of irradiation (IR). Olap pretreat;*Brca2*^*ko/ko*^ indicates *Brca2*^*ko/ko*^ mESC rescued by olaparib pretreatment. Scale bars, 10 μm. (**b**) Histogram showing gene-targeting efficiency. shPARP1-1/2; *Brca2*^*ko/ko*^ indicates *Brca2*^*ko/ko*^ mESC rescued by PARP1 knockdown. Actual numbers of targeted clones and total numbers of clones are indicated above each column. Inset shows Southern blot of the gene targeting.

BRCA2 is essential for the recruitment of RAD51 at DSB to mediate HR^1^,^21^. We therefore examined whether RAD51 recruitment in response to DSB induction was restored in the *Brca2*^*ko/ko*^ mESC rescued by PARP1 knockdown or by olaparib treatment. We did not observe any RAD51 foci in these cells in response to irradiation, although RAD51 was expressed at levels similar to the control cells (Fig. 2a and Supplementary Fig. 4a,b). Next, we used gene targeting to measure DSB-induced HR efficiency in the rescued cells^20^. We compared PL2F7 controls with or without PARP1 knockdown with two *Brca2*^*ko/ko*^ mESCs rescued by PARP1 knockdown. While control and PARP1 knockdown cells exhibited a targeting efficiency of 16.7–25%, *Brca2*^*ko/ko*^ rescued cells did not exhibit any targeted clones (Fig. 2b). Moreover, metaphase spreads revealed an increase in chromosomal aberrations in *Brca2*^*ko/ko*^ mESC rescued by PARP1 knockdown compared with the parental PL2F7 cells (Supplementary Fig. 4c,d). Together with the defect in RAD51 foci formation, these results demonstrate that DSB-induced HR is not restored in rescued *Brca2*^*ko/ko*^ mESC.

### Rescue of *Brca2* ^*ko/ko*^ haematopoietic cells by PARPi treatment

**Figure 3:**
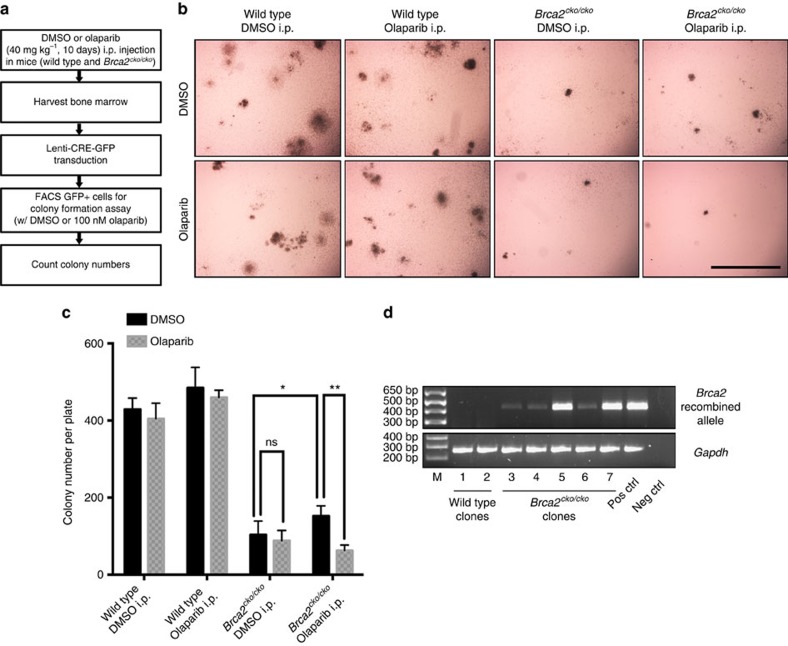
Rescue of *Brca2*^*ko/ko*^ haematopoietic cells by PARPi treatment in mice. (**a**) Workflow of clonogenic assay in haematopoietic progenitor cells. (**b**) Representative colony images of the clonogenic assay of haematopoietic progenitor cells. Scale bar, 2 mm. (**c**) Quantification on colony numbers of the clonogenic assay in haematopoietic progenitor cells. *P<0.05, **P<0.01 (*t*-test). (**d**) Genotyping of haematopoietic progenitor colonies from WT or *Brca2*^*cko/cko*^ mice receiving olaparib pretreatment. Primers are listed in Supplementary Note 1.

Given the pro-survival effects of PARP1 deficiency or PARP inhibition on the viability of *Brca2*^*ko/ko*^ mESC *in vitro*, we tested whether olaparib treatment *in vivo* can support survival of *Brca2*-deficient haematopoietic progenitor cells. Wild-type (WT) and *Brca2*^*cko/cko*^ mice were injected with either dimethylsulfoxide (DMSO) or olaparib for 10 days and then collected the bone marrow (Fig. 3a). We then transduced the bone marrow cells with CRE-GFP lentivirus to delete the conditional allele. Green fluorescent protein (GFP)-positive cells were sorted and cultured *in vitro* for colony formation. DMSO- and olaparib-pretreated WT cells formed an average of 428 and 485 colonies per plate, respectively. In contrast, the DMSO-pretreated *Brca2*^*cko/cko*^ cells formed very few and small colonies (average of 104 per plate). While olaparib-pretreated *Brca2*^*cko/cko*^ cells also formed small colonies, their number was significantly higher (average of 152 colonies per plate, *P*=0.0299, *t*-test) compared with DMSO-pretreated *Brca2*^*cko/cko*^ cells (Fig. 3b,c). In parallel, colony formation in the presence of 100 nM olaparib was also performed. The number of colonies from *in vivo* olaparib-pretreated *Brca2*^*cko/cko*^ cells was significantly reduced by *in vitro* olaparib post treatment, whereas the colony number in DMSO-pretreated *Brca2*^*cko/cko*^ cells remained unchanged (Fig. 3b,c). This suggests that the surviving olaparib-pretreated *Brca2*^*cko/cko*^ clones were indeed HR-defective and BRCA2-deficient, which was confirmed by genotyping (Fig. 3d).

### PARP1 deficiency protects degradation of replication forks

**Figure 4:**
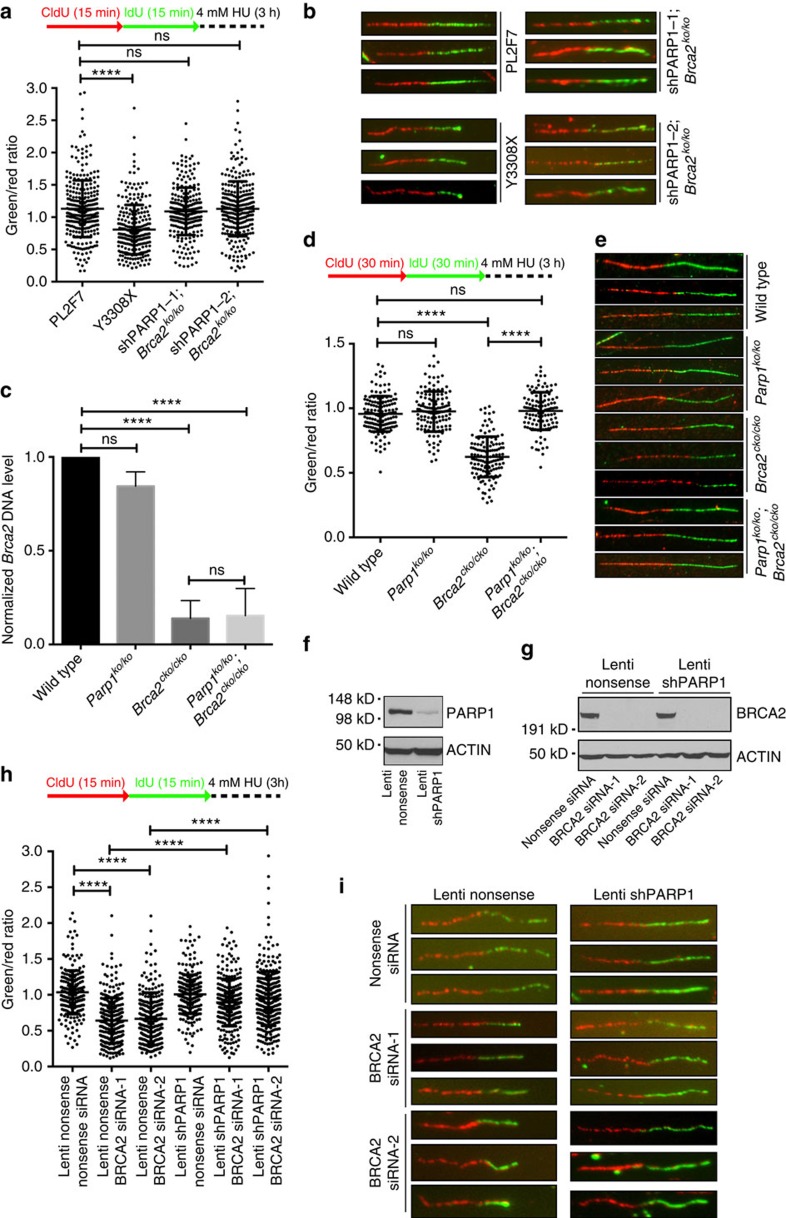
PARP1 deficiency protects BRCA2 loss-induced replication fork degradation. (**a**) Scattered plot showing DNA fibre analysis of indicated cell lines. ****P<0.0001, ns, not significant (Mann–Whitney test). (**b**) Representative images of DNA fibres as quantified in **a**. (**c**) qPCR showing the deletion of genomic *Brca2* DNA in B cells with the indicated genotypes. All the cells were infected by retrovirus-Cre. Primers are listed in Supplementary Note 1. ****P<0.0001, ns, not significant (*t*-test). (**d**) Scattered plot showing DNA fibre analysis in B cells. (**e**) Representative images of DNA fibres as quantified in **d**. (**f**) Western blot showing PARP1 level in MCF10A stable knockdown cells. (**g**) Western blot showing BRCA2 knockdown level by siRNA in PARP1 stable knockdown MCF10A clones. (**h**) Scattered plot showing DNA fibre analysis in MCF10A cells, ****P<0.0001 (Mann–Whitney test). (**i**) Representative images of DNA fibres quantified in **h**.

BRCA2 has been reported to protect replication forks from degradation, independent of its role in DSB repair by HR^2^. PARP1 has been shown to play important roles in the control of several aspects of replication fork dynamics, including fork restart, degradation and reversal^22^,^23^,^24^. Since cell viability is not associated with restoration of the HR defect, we therefore asked whether PARP1 deficiency conferred replication fork stability to *Brca2*^*ko/ko*^ mESC rescued by PARP1 knockdown. Integrity of the replication forks was examined using the DNA fibre assay, which allows single-molecule resolution of replication fork tracts^2^. Ongoing replication forks were labelled with chlorodeoxyuridine (CldU; red) and then with iododeoxyuridine (IdU; green) for 15 min each followed by fork stalling agent, hydroxyurea (HU) treatment for 3 h. We measured the track length of CldU- and IdU-labelled DNA fibres and calculated the IdU to CldU ratio. The ratio is close to 1 when the forks are protected, but <1 when the forks are degraded. While WT cells exhibited an average ratio of IdU to CldU tracts lengths close to 1, IdU tracts in mESC expressing a truncated allele of *BRCA2* (Y3308X) were considerably shorter (average IdU to CldU ratio=0.807, *P*<0.0001, Mann–Whitney test), indicating the degradation of nascent strands on replicative stress (Fig. 4a,b). Strikingly, in both *Brca2*^*ko/ko*^ clones rescued by PARP1 knockdown, IdU to CldU ratios were comparable to those observed in PL2F7 cells (average IdU to CldU ratio=1.09 and 1.12, respectively) (Fig. 4a,b). These results indicate that deficiency of PARP1 deficiency confers protection to the replication forks in the absence of functional BRCA2.

To determine whether the observed replication fork protection due to PARP1 deficiency is observed in other cell types, we also analysed replication fork integrity in mouse B lymphocytes. For reasons not well understood, unlike mESCs, B cells are able to tolerate loss of ‘essential' HR genes (for example, *BRCA1*, *BRCA2* and *CtIP*) with minimal impact on their growth *ex vivo*^25^,^26^. Nevertheless, these cells remain highly sensitive to agents that challenge replication forks^25^,^26^. We generated B lymphocytes from WT, *Parp1*^*ko/ko*^, *Brca2*^*cko/cko*^ and *Parp1*^*ko/ko*^*;Brca2*^*cko/cko*^ mice. The *cko* allele was deleted by transducing the cells with CRE-expressing retrovirus after stimulating B cells with lipopolysaccharide+interleukin-4 and RP105. Deletion of the *cko* allele was confirmed by quantitative PCR (Fig. 4c). On HU treatment, the average IdU to CldU ratios were determined to be 0.957, 0.976, 0.624 and 0.979 for WT, *Parp1*^*ko/ko*^ and *Brca2*^*ko/ko*^ and *Parp1*^*ko/ko*^*;Brca2*^*ko/ko*^ cells, respectively (Fig. 4d,e). Thus, consistent with our observation in mESC, loss of PARP1 resulted in protection of the nascent strand in *Parp1*^*ko/ko*^*;Brca2*^*ko/ko*^ B cells (*P*=0.1147 versus WT, Mann–Whitney test).

To test whether deficiency of PARP1 can rescue fork degradation induced by BRCA2 loss in human cells as well, we used immortalized human mammary epithelial cells MCF10A. Two independent clones with stable PARP1 knockdown were generated by transducing cells with shRNA-expressing lentivirus (Fig. 4f). We then knocked down BRCA2 by siRNA (Fig. 4g) and examined fork stability by DNA fibre assay. Average IdU to CldU ratios were 1.03, 0.64 and 0.66 in stable control clone with nonsense, BRCA2-1 and BRCA2-2 siRNA, respectively. However, the average ratios were 1.00, 0.89 and 0.95 in stable PARP1 knockdown clone with nonsense, BRCA2-1 and BRCA2-2 siRNA, respectively (*P*<0.0001, Mann–Whitney test) (Fig. 4h,i). The fact we did not observe fork degradation in MCF10A cells with stable PARP1 knockdown seems to contradict previous study in which fork degradation was observed in olaparib-treated BRCA2-proficient cells^24^. It is possible that fork protection is interfered by trapping of PARP1 by olaparib^12^, whereas in the PARP1 knockdown cells trapping does not occur. Alternatively, the level of BRCA2 in the reconstituted V-C8 cells is not the same as in heterozygous cells expressing a single copy of WT *BRCA2*, making the fork sensitive to olaparib. Taken together, our data suggest that PARP1 deficiency can rescue the BRCA2 loss-induced fork degradation in both mouse and human cells.

### Impaired MRE11 recruitment contributes to fork protection

**Figure 5:**
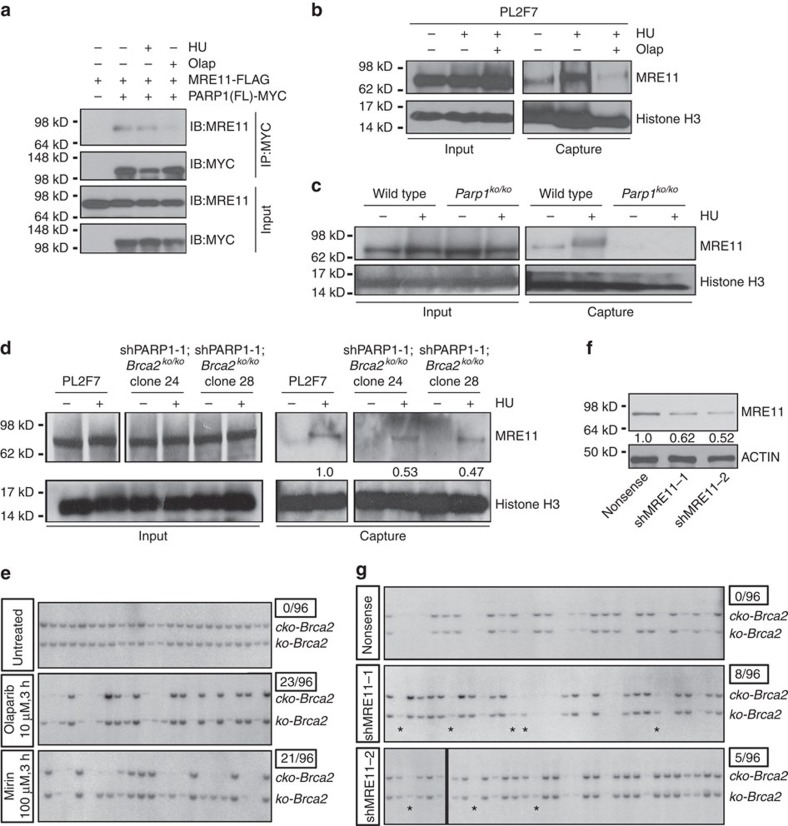
Impaired MRE11 recruitment contributes to fork protection and cell survival after BRCA2 loss. (**a**) Western blot of immunoprecipitation in HEK293T cells showing interaction of MRE11 and PARP1. (**b**) Western blot of iPOND samples in PL2F7 cells showing MRE11 and Histone H3 levels at the replication fork. HU, 4 mM, 4 h (same as below). Olaparib (Olap), 1 μM, 2 h pretreatment, with HU for 4 h. (**c**) Western blot of iPOND samples in MEF cells showing MRE11 and Histone H3 levels at the replication fork. (**d**) Western blot of iPOND samples from indicated cell lines showing MRE11 and Histone H3 levels at the replication fork. Numbers indicate quantification of relative band intensity of MRE11 normalized to Histone H3. These band intensities were compared with the intensity of MRE11 in PL2F7 cells, which was considered to be 1. (**e**) Representative Southern blot showing rescue of *Brca2*^*ko/ko*^ mESC by transient olaparib or mirin pretreatment. (**f**) Western blot showing MRE11 level in stable knockdown mESC clones. Numbers indicate quantification of relative band intensity of MRE11 normalized to corresponding ACTIN band intensity. These band intensities were compared with the intensity of MRE11 in Nonsense, which was considered to be 1. (**g**) Representative Southern blot showing rescue of *Brca2*^*ko/ko*^ mESC in two MRE11 stable knockdown clones. Asterisks indicate the rescued clones.

We next examined the molecular mechanism of the fork protection that may contribute to rescue of *Brca2*^*ko/ko*^ mESC. MRE11 nucleolytic activity is responsible for BRCA2 loss-induced fork degradation^2^,^24^. MRE11 is reported to interact with PARP1 (refs 22, 27). Interestingly, MRE11 has a PAR-binding RG or RGG-rich motif suggesting that it may be PARylated by PARP1 (refs 9, 27). We therefore investigated whether the effect of PARP1 deficiency was mediated via MRE11. Our data confirmed previous reports that MRE11 physically interacts with PARP1 independent of replication stress and presence of DNA; however, this interaction was disrupted when PARP was inhibited by olaparib (Fig. 5a and Supplementary Fig. 5d). We further identified the N-terminal region of PARP1 containing two Zn finger domains but not the catalytic domain to interact with MRE11. Our findings suggest that PARP1 and MRE11 interaction was dependent on PARP activity (Supplementary Fig. 5a–c). Since the interaction is mediated by PARP1 N-terminal instead of its catalytic domain, we also speculate that auto-PARylation of PARP1 may be important for PARP1 and MRE11 interaction. The fact that interaction between PARP1 N-terminal domain and MRE11 can also be disrupted by olaparib (Supplementary Fig. 5c) suggests that the N-terminal domain might be auto-PARylated.

Consistent with previous studies^2^, we observed rescue of the fork degradation in Y3308X *BRCA2*-expressing mESC by MRE11 inhibitor, mirin (Supplementary Fig. 6a), indicating MRE11 indeed mediates the fork degradation in BRCA2-deficient mESC.

To determine whether PARP inhibition or PARP1 deficiency affected recruitment of MRE11 to stalled replication forks, we examined the presence of MRE11 at the fork in mESC treated with HU (4 mM, 4 h) or HU with olaparib (1 μM, 2 h pretreatment followed by HU combination for another 4 h) by iPOND (isolation of proteins on nascent DNA)^28^. HU treatment induced marked increase in MRE11 recruitment to stalled replication forks. However, olaparib reduced the association of MRE11 to stalled replication forks (Fig. 5b). To examine the effects of PARP1 deficiency, we performed iPOND by using mESC with PARP1 knockdown (Supplementary Fig. 6e), as well as *Parp1*^*ko/ko*^-immortalized mouse embryonic fibroblasts (MEFs) (Fig. 5c and Supplementary Fig. 6b). Consistently, we observed a marked reduction of MRE11 recruitment in these PARP1-deficient cells. Furthermore, compared with PL2F7 cells, we detected a clear reduction in MRE11 recruitment at the fork in two independent *Brca2*^*ko/ko*^ mESC clones rescued by PARP1 knockdown (Fig. 5d and Supplementary Fig. 6c). On the basis of the above results, we conclude that impaired recruitment of MRE11 to stalled replication forks by PARP inhibition or PARP1 deficiency contributes to fork protection in *Brca2*^*ko/ko*^ mESC.

If fork protection indeed contributes to the rescue of *Brca2*^*ko/ko*^ mESC survival, it is difficult to reconcile the survival of *Brca2*^*ko/ko*^ mESC rescued by olaparib, because the olaparib-mediated inhibitory effect is transient. We therefore tested whether transient PARPi treatment was indeed sufficient to rescue *Brca2*^*ko/ko*^ mESC lethality. We pretreated PL2F7 cells with 10 μM olaparib for only 3 h and then performed the rescue experiment as described in Fig. 1a. Surprisingly, even such transient exposure to olaparib resulted in a number of viable *Brca2*^*ko/ko*^ mESC clones (24%) (Fig. 5e). This is in line with the iPOND analysis, which revealed that olaparib disrupts the MRE11 recruitment to the stalled fork (Fig. 5b). Unlike the *Brca2*^*ko/ko*^ mESC rescued by PARP1 knockdown, *Brca2*^*ko/ko*^ mESC rescued by olaparib resulted in fork degradation (Supplementary Fig. 6d,g). These cells also had significantly more breaks and gaps than the cells rescued by PARP1 knockdown (Supplementary Fig. 4c,d). These results suggest transient fork protection during the deletion of *Brca2* may contribute to rescue the survival of *Brca2*^*ko/ko*^ mESC, however, the subsequent survival of *Brca2*^*ko/ko*^ cells is not dependent on fork protection.

### Rescue of *Brca2* ^*ko/ko*^ mESC by MRE11 inhibition or deficiency

**Figure 6:**
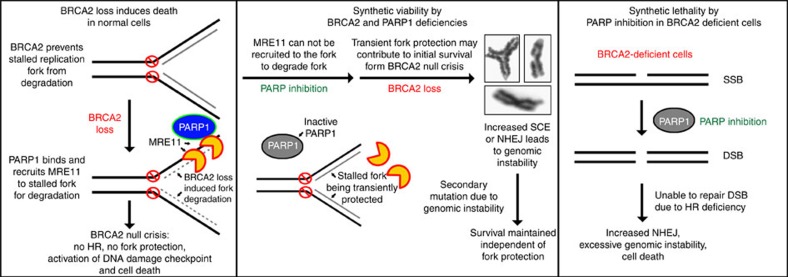
Cell model of synthetic viability between BRCA2 and PARP1 deficiency. Model to explain the difference between synthetic viability and synthetic lethality associated with BRCA2 and PARP1 loss. The outcome is dependent on the order of events. When BRCA2 loss is followed by PARP inhibition or PARP1 deficiency, it results in synthetic lethality (right). In contrast, when PARP inhibition or PARP1 deficiency is followed by BRCA2 loss, it results in synthetic viability (middle).

To directly test whether MRE11 inhibition can rescue the lethality of *Brca2*^*ko/ko*^ mESC, we pretreated PL2F7 cells with 100 μM mirin (the same dosage that can rescue fork degradation as shown in Supplementary Fig. 6a) for 3 h and then performed the rescue experiment described in Fig. 1a. We obtained a number of *Brca2*^*ko/ko*^ mESC (22%, Fig. 5e). Like other BRCA2-null rescued mESC described above, these cells lacked irradiation-induced RAD51 foci (Supplementary Fig. 6f,h), indicating that HR was not restored. To rule out any off-target effects of mirin, we generated MRE11 stable knockdown clones by shRNA in PL2F7 cells (Fig. 5f). Consistent with the rescue by mirin, MRE11 stable knockdown resulted in the survival of *Brca2*^*ko/ko*^ mESC as evident in two independent MRE11 knockdown clones (Fig. 5g). Although we cannot rule out the possibility that other genetic and epigenetic alterations or effects on cell cycle regulation induced by PARP or MRE11 inhibition or knockdown may contribute to viability, our findings suggest that fork protection may play an important role in rescue of cellular viability. We also conclude that while transient fork protection by either PARPi or MRE11 inhibition contributes to the initial survival of *Brca2*^*ko/ko*^ mESC, it is not essential to maintain the growth of these cells (Fig. 6).

### *Brca2^cko/cko^* mice are tumour prone in a *Parp1^*ko/+*^* background

**Figure 7:**
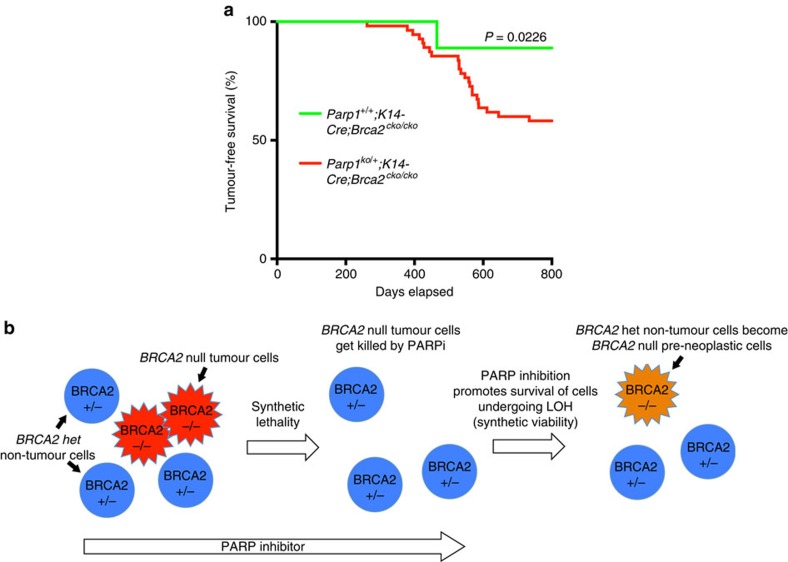
*Brca2^*cko/cko*^* mice with *K14-Cre* are tumour prone in a *Parp1^ko/+^* background. (**a**) Kaplan–Meier curves showing tumour-free survival percentage of *K14-Cre; Brca2*^*cko/cko*^ mice under *Parp1*^*+/+*^ or *Parp1*^*ko/+*^ genetic background. (**b**) Model to explain the targeting of BRCA2-deficient tumours by PARPi and the potential concern related to its role in cell survival.

It is known that mESC lethality due to BRCA1 loss can be rescued by 53BP1 loss, which restores end resection and HR, and suppresses tumorigenesis in mice^25^,^29^. In contrast PARP1 deficiency does not restore HR but results in fork protection. Although the fork protection promotes genomic stability relative to cells that lack fork protection (Supplementary Fig. 4c,d), the level of genomic instability is higher than the cells expressing WT BRCA2. We predict that viable cells exhibiting genomic instability are likely to contribute to tumorigenesis. To directly test whether PARP inhibition or PARP1 deficiency can facilitate tumorigenesis in BRCA2-deficient mice, we generated *K14-Cre;Brca2*^*cko/cko*^ mice on *Parp1*^*+/+*^ and *Parp1*^*ko/+*^ genetic backgrounds. We observed a marked increase in the incidence of epithelial tumours in *K14-Cre;Brca2*^*cko/cko*^ mice in a *Parp1*^*ko/+*^ genetic background (23 out of 56 mice, 41.1%) compared with that in *Parp1*^*+/+*^ genetic background (2 out of 18 mice, 11.1%; *P*=0.0226, Fisher's exact test; Fig. 7a, Supplementary Fig. 7 and Supplementary Table 1), suggesting PARP1 deficiency indeed facilitates BRCA2 loss-induced tumorigenesis.

## Discussion

PARylation by PARP1 affects the cellular localization and site-specific recruitment, such as recruitment to DNA damage sites, of a number of proteins involved in a wide-range of biological processes^8^. Here we have investigated the functional consequence of PARP inhibition on HR-proficient cells that express functional BRCA2. PARPi is toxic to BRCA2-deficient cells, however, we show that PARP inhibition can paradoxically promote their survival. We show that knockdown or heterozygous loss of *Parp1*, as well as olaparib treatment before BRCA2 loss rescues the lethality of *Brca2*^*ko/ko*^ mESC. We speculate that the order of two genetic events, PARP inhibition or PARP1 deficiency and BRCA2 loss, dictates the outcome of either synthetic lethality or synthetic viability (Fig. 6). Inactivation of PARP1 before loss of BRCA2 is likely to promote survival by preventing MRE11-mediated toxicity linked to DNA replication and subsequent cell death. In contrast, in BRCA2-null cells, MRE11 may persist at the replication forks and degrade it, causing persistent DNA damage. This genomic instability would be exacerbated and result in cell death by the subsequent PARPi due to the additional block in single-strand break repair or perhaps due to the PARP1 trapping^12^,^30^.

The role of MRE11 in cell viability by PARP inhibition or PARP1 deficiency is demonstrated by the rescue of *Brca2*^*ko/ko*^ mESC by knockdown of *MRE11*, as well as by treatment of cells with mirin before BRCA2 loss. Although our findings suggest that the MRE11-mediated fork protection by PARP inhibition or PARP1 deficiency may contribute to the rescue of *Brca2*^*ko/ko*^ mESC, is not essential for maintaining cell survival even in the absence of HR. For example, a short treatment of olaparib or mirin rescues *Brca2*^*ko/ko*^ mESC. What contributes to the viability in this case is puzzling because the rescued cells are unable to protect the fork in response to replication stress. We hypothesize that when PARP or MRE11 are inhibited and the conditional allele of *Brca2* is deleted, cells are able to overcome the BRCA2-null crisis (that is, BRCA2 loss-induced excessive fork degradation, fork collapse resulting in un-repairable DSBs causing cell death) because the replication forks are transiently protected due to defect in MRE11 recruitment. We propose that this transient fork protection may be sufficient for the initial survival. Once the cells survive the BRCA2-null crisis, their survival is no longer dependent on PARP inhibition or fork protection (Fig. 6). These results therefore suggest that the rescued cells may acquire epigenetic changes or secondary mutations perhaps through mutagenic non-homologous end joining or sister chromatid exchanges, which are indeed enhanced by PARP inhibition or PARP1 deficiency (Supplementary Fig. 4c–e)^13^,^31^,^32^.

Interestingly, in an independent study, MRE11 recruitment to nascent DNA strands was shown to be dependent on PTIP and the associated histone methyltransferases MLL3 and MLL4 (ref. 33). Similar to PARP1 deficiency, PTIP loss inhibits degradation of stalled forks and rescues the viability of *Brca2*^*ko/ko*^ mESC. An important functional consequence of fork protection associated with PTIP loss is resistance to PARPi and cisplatin in *Brca2*-deficient tumour cells that is independent of the presence of secondary reversion mutations. Similar association between fork protection and PARPi or cisplatin resistance was also observed with CHD4 loss^33^,^34^. Together, this study and our present study highlight the importance of fork protection in rescuing BRCA2 loss-associated phenotypes without restoration of HR.

Our ability to rescue the lethality of *Brca2*^*ko/ko*^ cells may have important implications. We predict that in addition to killing the tumour cells, PARPi may facilitate the survival of normal *BRCA2* heterozygous cells that undergo loss of heterozygosity. These cells that will otherwise undergo apoptosis may be able to survive and have the potential to become neoplastic (Fig. 7b). Taken together, our findings provide evidence that PARPi, although lethal to HR-deficient cancers, is not innocuous in HR-proficient normal cells.

## Methods

### mESC culture

All mESCs were cultured on mitotically inactive SNL feeder cells in M15 media, which is Knockout DMEM media (Life Technologies) supplemented with 15% fetal bovine serum (FBS; Life Technologies), 0.00072% beta-mercaptoethanol, 100 U ml^−1^ penicillin, 100 μg ml^−1^ streptomycin and 0.292 mg ml^−1^ L-glutamine at 37 °C, 5% CO_2_. PL2F7 cells were generated from AB2.2 mouse embryonic stem cell line by knocking out one copy of *Brca2* and flanking the other allele of *Brca2* with two *LoxP* sites^20^.

### Generation of PARP1 stable knockdown mESC clones

Two different shRNAs against mouse *Parp1* mRNA and one control shRNA (nonsense) were cloned into pSUPERIOR.retro.neo vector (Oligoengine) into BglII and HindIII restriction sites. shRNA sequences are listed in Supplementary Note 1. shRNA vectors were linearized by ScaI, 20 μg of the linearized vectors were electroporated into 1 × 10^7^ mESCs suspended in 0.9 ml PBS using Gene Pulser (Bio-Rad) at 230 V, 500 μF. After electroporation, 2 × 10^4^ cells were plated into 10 cm plate, and G418 selection (0.18 mg ml^−1^) was started 24 h after electroporation. G418 was withdrawn after 5 days of selection for colonies to become visible. Individual colonies were picked into 96-well plate and PARP1 knockdown level of each clone was determined by western blot.

### Generation of *Parp1* knockout clones in mESC by CRISPR-Cas9n

Small guide RNA (sgRNA) was designed within exon 2 of mouse *Parp1* gene by using CRISPR design tool at http://crispr.mit.edu. The oligonucleotide sequences are listed in Supplementary Note 1. Oligonucleotides were cloned into Cas9 nickase (Cas9n) expression vector pX335-U6-Chimeric_BB-CBh-hSpCas9n (D10A) (Addgene, plasmid #42335). Oligonucleotides were cloned into the vector to generate sgRNAs. SURVEYOR nuclease assay was performed by transfecting sgRNA into NIH3T3 cells and PCR amplifying the edited genomic region. Nuclease S was used to digest the re-annealed PCR products^35^. Primers for SURVEYOR nuclease assay are listed in Supplementary Note 1.

For sgRNA transfection in mESC, 3 × 10^5^ mESCs were plated in 6-well plates 24 h before transfection. sgRNAs (1 μg of each) were co-transfected with pcDNA3.1 (1 μg) plasmid (Life Technologies) by using 12 μl FuGENE 6 transfection reagent (Promega). Cells were trypsinized and 1 × 10^4^ cells were re-plated into 10 cm plate 24 h after transfection. G418 selection (0.18 mg ml^−1^) was started 24 h after re-plating. G418 selection was performed for 5 days followed by growth in M15 media for colonies to become visible. Individual colonies were picked into 96-well plate and PCR was performed to amplify the targeted region. PCR primers are listed in Supplementary Note 1. PCR products of clones having band sizes different from WT band were purified and sequenced. PARP1 protein expression of each clone was determined by western blot.

### Deletion of *Brca2 cko* allele and selection of *Brca2* ^*ko/ko*^ mESC

A unit of 20 μg of PGK-Cre plasmid DNA were electroporated into 1 × 10^7^ mESCs suspended in 0.9 ml PBS by Gene Pulser (Bio-Rad) at 230 V, 500 μF. HAT selection was started 36 h after electroporation and lasted for 5 days, followed by selection in HT media for 2 days and then normal M15 media until colonies became visible. Colonies were picked into 96-well plate. For extracting genomic DNA, colonies were lysed in 50 μl mESC buffer (10 mM Tris-HCl (pH 7.4), 10 mM EDTA, 10 mM NaCl, 5 mg ml^−1^ sodium lauroyl sarcosinate, 1 mg ml^−1^ proteinase K) at 55 °C overnight, and DNA was precipitated by 100 μl 75 mM NaCl in absolute ethanol. Genomic DNA was rinsed by 70% ethanol and digested by EcoRV at 37 °C overnight for Southern blot.

### Southern blot

EcoRV-digested DNA was electrophoresed on 1% agarose gel in 1 × TBE (0.1 M Tris, 0.1 M boric acid and 2 mM EDTA, pH 8.0) and transferred to nylon membrane. DNA probe for distinguishing conditional *Brca2* allele (*cko-Brca2*, 4.8 kb) and *Brca2* knockout allele (*ko-Brca2*, 2.2 kb)^20^ was labelled by [α-^32^P]-dCTP by Prime-It II Random Primer Labeling Kit (Agilent Technologies) and hybridized with Hybond-N+ nylon membrane (GE Healthcare) at 65 °C overnight. Membrane was washed twice with saline sodium citrate phosphate (SSCP) buffer containing 0.1% SDS in and exposed in phosphor image screen overnight and subsequently developed in Typhoon image scanner.

### Mice and tumour pathology

*Brca2*^*ko/+*^ mice carrying a null allele of *Brca2* and *CD19-Cre* mice expressing Cre transgene under the control of CD18 promoter were used^21^,^36^. *K14-Cre* and *Brca2*^*cko/cko*^ mice were obtained from NCI-Frederick mouse repository (strain number:01XF1 and 01XB9, respectively)^37^, *Parp1*^*ko/ko*^ mice were obtained from Jackson Laboratory (stock number: 002779)^11^. All genotyping primers are listed in Supplementary Note 1. These mice were crossed to generate mice of desired genotypes. Mice of each genotype were randomly selected for experiments. Mice necropsy was performed by NCI-Frederick Pathology and Histotechnology Laboratory. Tumours were evaluated by blindly a board-certified veterinary pathologist, who was blinded to the group allocation.

All animal studies were performed as per the guidelines outlined in the Guide for the Care and Use of Laboratory Animals and protocols approved by the NCI-Frederick Animal Care and Use Committee.

### Embryo dissection and laser capture microdissection

Timed pregnancy in mice was set-up and embryos were collected at E8.5 or E10.5. Embryos were dissected according to established protocol^38^. Dissected embryos were lysed in 50 μl mESC lysis buffer at 55 °C overnight. Genomic DNA was precipitated using 3 M sodium acetate (1/10 volume) and 2 volumes of ethanol. Precipitated DNA was rinsed with 70% ethanol, resuspended in 1 × TE buffer and used for genotyping PCR. Genotyping PCR primers are listed in Supplementary Note 1.

For laser capture microdissection (LCM), whole embryos were fixed in 10% neutral buffered formalin solution (Sigma). Embryos were embedded in paraffin and were serially sectioned at 5 μm. The section adjacent to haematoxylin and eosin was mounted on LCM slide (MMI CellCut Plus from Molecular Machines & Industries) for embryo collection. LCM workflow, LCM slide preparation, target dissection and collection were carried following standard procedures^39^,^40^. Collected embryos were incubated in 25 μl DNA lysis buffer (Arcturus PicoPure DNA extraction Kit, Life Technologies) for 48 h at 55 °C. Genomic DNA was precipitated by adding 1 μl glycogen (Sigma), 3 M sodium acetate (1/10 volume) and 2 volumes of ethanol. DNA was precipitated at −20 °C for at least 1.5 h. Precipitated DNA was rinsed with 70% ethanol, resuspended in 1 × TE buffer and used for genotyping PCR. Genotyping PCR primers were listed in Supplementary Note 1.

### B-cell culture

Resting splenic B cells were isolated from 8- to 12-week-old mice with anti-CD43 microbeads (anti-Ly48; Miltenyi Biotech) and were cultured with lipopolysaccharide (25 μg ml^−1^; Sigma) and interleukin-4 (5 ng ml^−1^; Sigma) and RP105 (0.5 μg ml^−1^; BD)^41^. Stimulated B cells were infected with retrovirus-CRE to ensure high level of deletions of *Brca2* in these cells.

### MEF isolation

MEF cells were isolated from 13.5-day embryos from WT and *Parp1*^*ko/ko*^ mice. Head and liver were removed and remaining tissues were digested by 0.05% trypsin for 10 min. Embryo tissue was forced through 21 G needle and was pipetted three times for dissociation. Cells were then plated and cultured in high-glucose DMEM with 10% FBS, 100 U ml^−1^ penicillin, 100 μg ml^−1^ streptomycin and 0.292 mg ml^−1^ L-glutamine at 37 °C, 5% CO_2_, 3% O_2_. MEF cells were immortalized by SV40 retroviral infection.

### *PARP1* and *BRCA2* knockdown in MCF10A cells

Immortalized human mammary epithelial MCF10A cell line is a kind gift from Dr Esta Sterneck (NCI-Frederick, NIH). Cells were maintained in DMEM-F12 media (Life Technologies) containing 10% FBS, 100 U ml^−1^ penicillin, 100 μg ml^−1^ streptomycin, 0.292 mg ml^−1^ L-glutamine, 10 μg ml^−1^ insulin, 100 ng ml^−1^ choleratoxin, 20 ng ml^−1^ epidermal growth factor and 0.5 μg ml^−1^ hydrocortisone at 37 °C, 5% CO_2_. Lentiviral shRNA vector against human *PARP1* (TRCN0000007930) and control shRNA (SHC202) was purchased from Sigma (MISSION shRNA). For packaging lentivirus, HEK293T cells (American Type Culture Collection) at 80% confluency were co-transfected with shRNA vector, pRSV-Rev, pMDLg-pRRE and pHCMVG. Packaging vectors were kind gifts from Dr Steven Hou (NCI-Frederick, NIH). After 72 h transfection, supernatant was collected and 0.45 μm filtered before being used for infecting MCF10A cells. After 48 h infection, MCF10A cells were subjected to 3.3 μg ml^−1^ puromycin selection for 2 days. Puromycin-resistant cells were expanded and tested for PARP1 expression. Two siRNAs against human *BRCA2* were purchased from Dharmacon (siGenome D-003462-01 and D-003462-02) and were transfected into PARP1 stable knockdown MCF10A cells by Lipofectamine 2000 (Life Technologies). After 48 h transfection, cells were used for DNA fibre assay, as well as for analysing BRCA2 protein expression by western blot.

### iPOND

To perform iPOND, 1.5 × 10^8^ cells were labelled with 10 μM EdU (Life Technologies) and treated with 4 mM HU for 4 h as indicated^42^. Cells were crosslinked with 1% formaldehyde for 10 min at room temperature, quenched with 0.125 M glycine and washed with PBS. For the conjugation of EdU with biotin azide, cells were permeabilized with 0.25% Triton X-100 in PBS buffer, and incubated in click reaction buffer (10 mM sodium-L-ascorbate, 20 μM biotin azide (Life Technologies) and 2 mM CuSO_4_) for 2 h at room temperature. Cells were resuspended in lysis buffer (50 mM Tris-HCl (pH 8.0) and 1% SDS) supplemented with protease inhibitors (Roche), and chromatin was solubilized by sonication in a Bioruptor (Diagenode-Pico) at 4 °C for 24 min. After centrifugation, supernatants were incubated for 1 h streptavidin-MyOne C1 beads (Life Technologies). Beads were washed and captured proteins were eluted by boiling beads in 2 × NuPAGE LDS Sample Buffer (Life Technologies) containing 200 mM dithiothreitol for 40 min at 95 °C. Proteins were resolved by electrophoresis using NuPAGE Novex 4–12% Bis-Tris gels and detected by western blot with the indicated antibodies.

### HR assay

HR assay was performed by gene-targeting assay^20^. Briefly, mESCs were electroporated by the targeting vector and were selected in hygromycin. Colonies were picked and targeted clones were identified by Southern hybridization.

### DNA fibre assay

Cells were sequentially pulsed by 8 μg ml^−1^ CldU for 15 min for mESC and MCF10A cells, or 30 min for B cells followed by 90 μg ml^−1^ IdU for 15 min for mESC and MCF10A cells, or 30 min for B cells. Cells were then treated with 4 mM HU for 3 h before they were resuspended in PBS. A volume of 3 μl of cell suspension containing ∼3 × 10^5^ cells were mixed with 7 μl lysis buffer (200 mM Tris-HCl (pH 7.4), 50 mM EDTA, 0.5% SDS) on glass slides and incubated at room temperature for 8 min before DNA fibre was spread. Fibres were fixed in methanol and acetic acid (3:1) at 4 °C overnight, rehydrated by PBS and denatured in 2.5 M HCl for 1 h. After rinsing away HCl by PBS, slides were blocked in PBS with 5% bovine serum albumin (BSA) for 1 h and incubated with anti-BrdU antibody (Mouse, #347580, Becton Dickinson, 1:100 dilution) and anti-BrdU antibody (Rat, ab6326, Abcam, 1:500 dilution) at 4 °C overnight. Slides were rinsed with PBS with 0.1% Tween-20 (PBST) and incubated with AlexaFluo488-conjugated anti-mouse IgG secondary antibody and AlexaFluo594-conjugated anti-rat IgG secondary antibody for 1 h at room temperature. Slides were rinsed with PBST and mounted by mounting media (Prolong Gold, Invitrogen). Images were taken in Zeiss Axio Imager Z1 microscope and fibre length was measured by ImageJ software (NIH).

### Protein expression and interaction studies

The following antibodies were used: anti-PARP1 (Rabbit, #9542, Cell Signaling Technology); anti-PARP2 (Rabbit, ab93416, Abcam); anti-GAPDH (Rabbit, #2118, Cell Signaling Technology); anti-ACTIN (Goat, sc-1616, Santa Cruz); anti-RAD51 (Rabbit, PC130, Calbiochem); anti-γH2AX (Mouse, 05–636, Millipore); anti-pS317 CHK1 (Rabbit, #12302, Cell Signaling Technology); anti-CHK1 (Mouse, sc-8408, Santa Cruz); anti-MRE11 (Goat, sc-5859, Santa Cruz); anti-MRE11 (Rabbit, Callen and Nussenzweig, unpublished); anti-MYC (Mouse, 631206, Clontech); anti-MYC (Rabbit, 2272, Cell Signaling Technology); anti-p53 (Mouse, 2524, Cell Signaling Technology); anti-pS15 p53 (Rabbit, 9284, Cell Signaling Technology); anti-Histone H3 (Rabbit, 9715, Cell Signaling Technology); anti-PCNA (Rabbit, 13110, Cell Signaling Technology); anti-p19ARF (Rabbit, ab80, Abcam); and anti-BRCA2 (Mouse, OP95, Millipore). For western blot, cells were lysed in SDS lysis buffer (2% SDS, 10% glycerol, 0.1 M dithiothreitol and 0.2 M Tris-HCl (pH6.8)) and subjected to SDS–PAGE gel electrophoresis, and subsequently transferred to nitrocellulose membrane. Blots were incubated with the indicated primary antibodies at 4 °C overnight, washed by PBST and probed with corresponding horseradish peroxidase-conjugated secondary antibodies at room temperature for 2 h and subjected to ECL (Amersham). For immunofluorescence, cells were fixed with 4% paraformaldehyde, permeabilized by 0.25% Triton X-100 and blocked with 3% BSA. Cells were incubated with the indicated primary antibodies at 4 °C overnight. After washing four times with PBST, cells were incubated with AlexaFluo488-conjugated anti-rabbit IgG antibody and AlexaFluo568-conjugated anti-mouse IgG antibody (Life Technologies) at room temperature for 2 h. Nucleus was counterstained by 4,6-diamidino-2-phenylindole. Images were taken on Zeiss LSM 510 confocal microscope.

For immunoprecipitation, cells were lysed in IP buffer (30 mM Tris-HCl (pH 8.0), 75 mM NaCl, 10% glycerol and 0.5% Triton X-100) containing protease inhibitors (Complete Mini protease inhibitor cocktail tablets, Roche) at 4 °C for 15 min and subjected to centrifugation at 15,000*g* for 15 min at 4 °C. Supernatant was transferred and added 30 μl beads (Protein G sepharose 4 Fast Flow, GE Healthcare) together with 2 μg indicated antibodies and incubated on rocker at 4 °C for 4 h. Beads were rinse four times by IP buffer at 800*g* for 1 min each time. Proteins were dissociated from the beads by SDS lysis buffer and boiled at 95 °C for 10 min. For the immunoprecipitation with DNase treatment, DNase I (2 U μl^−1^, New England Biolabs) was added to the IP buffer with the EDTA-free protease inhibitors (Complete EDTA-free protease inhibitor cocktail tablets, Roche) and IP procedures were the same as mentioned above. Uncropped images of western blot are shown in Supplementary Fig. 8.

### Generation of constructs expressing mouse PARP1 fragments

Mouse full-length *Parp1* cDNA and all other fragments (N, M, WGR and CAT)^43^ were PCR amplified from MGC mouse *Parp1* cDNA clone (GE Healthcare, clone ID: 2648390) and was subcloned into pcDNA3.1 (+) vector and fused with a MYC tag in the C terminus. PCR primers were listed in Supplementary Note 1. For immunoprecipitation, HEK293T cells were used for transfection. All groups were transfected by MRE11-Flag plasmid (a kind gift from Dr John Petrini, MSKCC), together with pcDNA3.1 (+) empty vector or full-length or other fragments of PARP1 by Lipofectamine 2000. After 48 h transfection, immunoprecipitation was performed by pulling down with anti-MYC antibody (Mouse, 631206, Clontech), subsequent detection by western blot was performed by indicated antibodies.

### Haematopoietic stem cell assay

WT and *Brca2*^*cko/cko*^ mice (two mice per group) were injected either by DMSO or 40 mg kg^−1^ olaparib (Selleckchem) for 10 days before their bone marrow was collected from the femur and tibia. Bone marrow was cultured in DMEM media containing 10% FBS, 100 ng ml^−1^ murine stem cell factor, 100 ng ml^−1^ human thrombopoietin, 100 ng ml^−1^ human Flt-3 ligand (hFlt-3L) and 50 ng ml^−1^ murine interleukin-6. Bone marrow cells were transduced by lentivirus expressing CRE-ires-GFP. For lentivirus packaging, HEK293T cells were transfected by lentiviral CRE-ires-GFP vector together with HIV gag-pol and VSVG vectors. After 48 h transfection, viral supernatant was collected and 0.45 μm filtered and was added to RetroNectin (25 μg ml^−1^, T100B, Takara)-coated six-well plates. Plates were centrifuged at 2,600*g* for 2 h at 4 °C in order for the virus to attach. Supernatant was removed and plates were ready to be used for transducing bone marrow cells. Three hours after bone marrow was collected from the mice, bone marrow cells were added to the lentiviral plates. Bone marrow cells were transferred every 12 h to fresh lentiviral plates to achieve higher transduction efficiency. Four rounds of transduction were performed before the GFP-positive cells were subjected to sorting on BD FACSAria II cell sorter. Immediately after sorting, the transduced GFP-positive bone marrow cells were plated in MethoCult (StemCell Technologies, Vancouver, Canada) as per the manufacturer's instructions. The following haematopoietic growth factors were added: 100 ng ml^−1^ murine stem cell factor; 100 ng ml^−1^ human thrombopoietin; 100 ng ml^−1^ human Flt-3 ligand (hFlt-3L); 50 ng ml^−1^ murine interleukin-6 and 30 ng ml^−1^ murine interleukin-3 (Peprotech Inc. Rocky Hill, NJ, USA). DMSO or olaparib (100 ng ml^−1^) was also added. Cells were plated at a density of 1.375 × 10^4^ cells per ml per plate in 35 mm cell dishes (Nunc) in triplicates and were incubated at 37 °C, 5% CO_2_ for 7–10 days. In all, 15–20 random images per plate were taken under × 2 objective and colony number was counted manually. Average colony number per image was calculated and was multiplied by 50 to get the total colony number per plate. For genotyping the colonies, individual colonies were picked and lysed by the mESC buffer as mentioned above at 55 °C overnight. Genomic DNA was precipitated by adding 1 μl glycogen, 3 M sodium acetate (1/10 volume) and 2 volumes of ethanol. DNA was precipitated at −20 °C for at least 1.5 h. Precipitated DNA was rinsed with 70% ethanol, resuspended in water and used for genotyping PCR. Genotyping PCR primers were listed in Supplementary Note 1.

### Cell cycle analysis

Cells were trypsinized, resuspended and fixed in 70% ethanol at 4 °C for 30 min. Cells were then rinsed by PBS, resuspended and incubated in PI and RNase staining buffer (BD) at 37 °C for 30 min. Cell cycle distribution was analysed in BD FACSCalibur flow cytometer.

### Karyotyping of mESC

The metaphases were arrested by incubation with Colcemid (KaryoMax Colcemid Solution, Invitrogen, Carlsbad, Calif., USA) (10 μg ml^−1^) 3 h before harvest. Cells were collected and treated with hypotonic solution (KCl 0.075 M) for 15 min at 37 °C and fixed with methanol:acetic acid 3:1. Slides were prepared and chromosomal aberrations were analysed.

### Quantitative PCR

qPCR to determine *Brca2* genomic deletion in B cells was performed by using iTaq Universal SYBR Green Supermix (Bio-Rad). qPCR reaction was run on Mx3000P qPCR system (Agilent Technologies). Primers are listed in Supplementary Note 1.

### Statistics

Statistics was performed by two-tailed *t*-test, Mann–Whitney test, two-tailed Fisher's exact test or *χ*^2^-test unless otherwise specified. All error bars represent s.d. Statistical tests were justified appropriate for every figure. The data are normally distributed, and the variance between groups that are being statistically compared is similar. *P*<0.05 was considered statistically significant. No statistical methods or criteria were used to estimate sample size or to include or exclude samples. The investigators were not blinded to the group allocation during the experiments unless otherwise specified.

### Data availability

This is to confirm that all relevant data are available from the authors on request.

## Additional information

**How to cite this article:** Ding, X. *et al.* Synthetic viability by BRCA2 and PARP1/ARTD1 deficiencies. *Nat. Commun.* 7:12425 doi: 10.1038/ncomms12425 (2016).

## Supplementary Material

Supplementary Information Supplementary Figures 1-8, Supplementary Table 1 and Supplementary Note 1
