# Supplementary material for: Synthetic viability by BRCA2 and PARP1/ARTD1 deficiencies
Source: Nat Commun. 2016 Aug 8;7:12425. doi: 10.1038/ncomms12425 (PMC4979061; doi:10.1038/ncomms12425)
Supplement: Supplementary Information — Supplementary Figures 1-8, Supplementary Table 1 and Supplementary Note 1 [file ncomms12425-s1.pdf]

Supplementary Information

Supplementary Figure 1 | Impact of PARP inhibition/PARP1 deficiency on CHK1 activation, cell cycle progression and p53/p19ARF response. **a**, Western blot showing CHK1 activation in PL2F7 cells treated with HU and olaparib. HU, 4 mM, 3 hours. Olaparib (Olap), difference doses, 3 hours. **b**, Schematic representation of mESC model. *HP* and *RT* represent 5′ and 3′ halves of human *HPRT1* minigene. **c**, Cell cycle profile of PL2F7 cells treated with olaparib. **d**, Quantification on the percentage of cells in different cell cycle stages as in (**c**). **e**, Western blot showing PARP1 level in mESC stable knockdown clone. **f**, Cell cycle profile of PARP1 stable knockdown mESC clone. **g**, Quantification on the percentage of cells in different cell cycle stages as in (**f**). **h**, Western blot showing p53 response under HU and olaparib treatment in PL2F7 cells. HU, 4 mM, 3 hours. Olaparib, 10 μM, 3 hours.

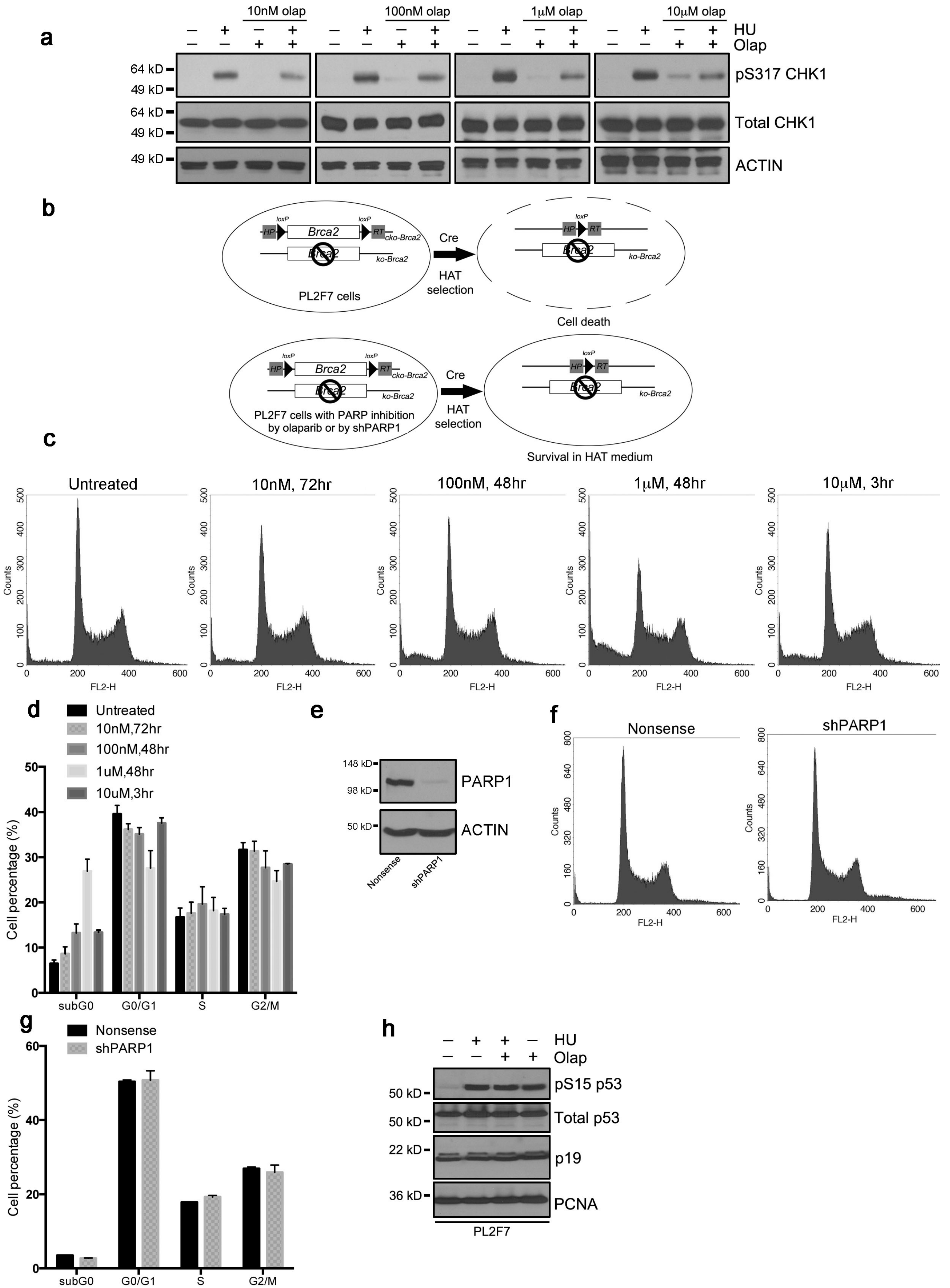

Supplementary Figure 1

Supplementary Figure 2│Knockout *Parp1* in mESC by using CRISPR/Cas9n. a, Mouse *Parp1* exon2 sequence, sgRNA pairs (red) and their PAM sequences (green). **b**, Surveyor assay showing efficacy of sgRNA pairs-guided Cas9n cleavage in NIH3T3 cells. All used oligonucleotides are listed in **Supplementary Note 1**. **c, d, e**, Chromatogram (**c, d**) and sequences (**e**) of *Parp1* heterozygous (het) clone (clone 1) and *Parp1* null clone (clone 21) in PL2F7 cells by using the sgRNA pairs. Clone 21 is a compound het clone. **f**, Western blot showing PARP1 protein level in wild-type, *Parp1* het and null clones. **g**, Southern blot showing the rescue of $Brca2^{ko/ko}$ mESC in the *Parp1* het clone (clone 1).

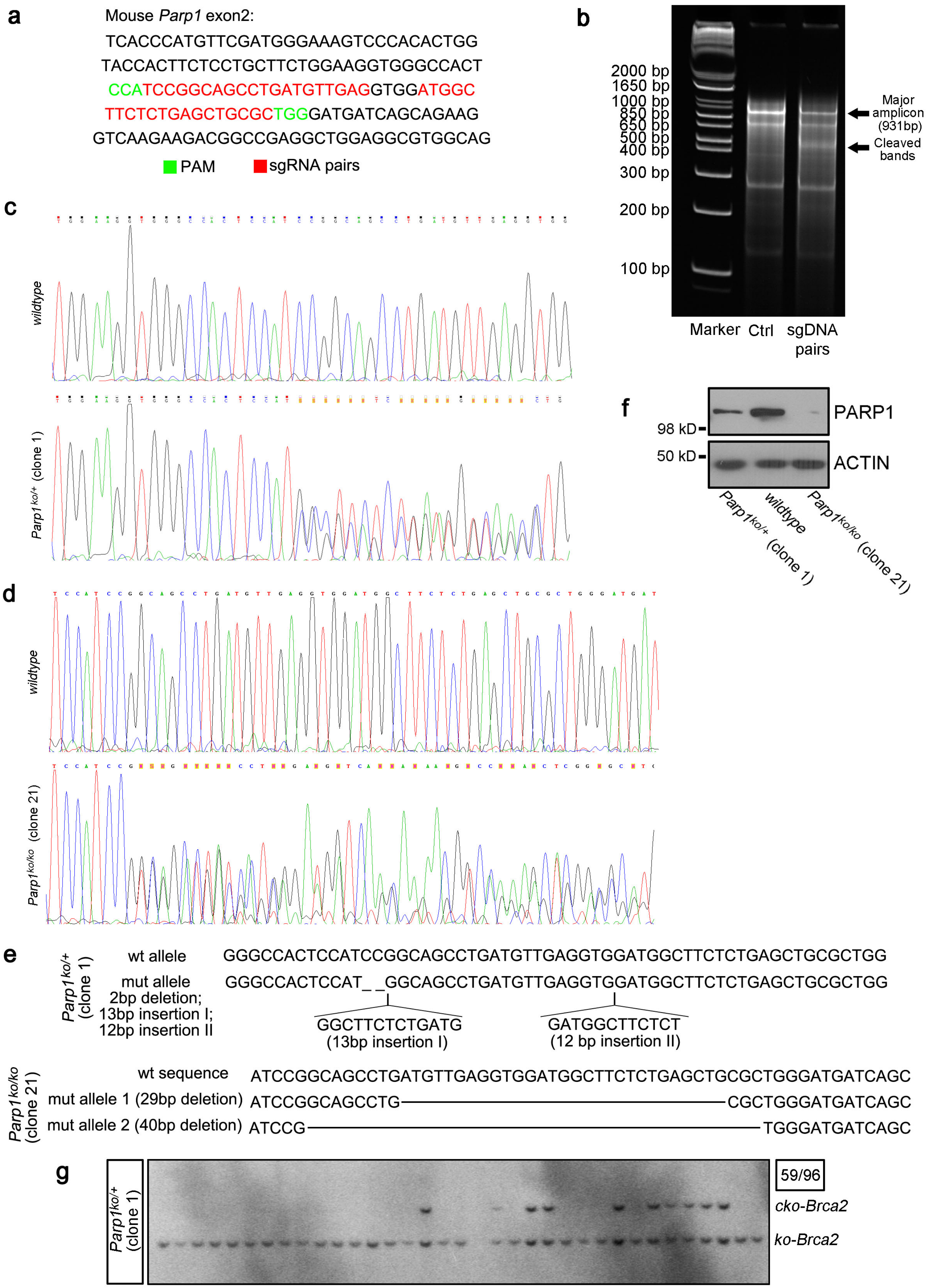

Supplementary Figure 2

Supplementary Figure 3│*Parp1* deficiency partially rescues *Brca2*$^{ko/ko}$ mouse embryos. **a and b**, Table showing numbers of E8.5 embryos with indicated genotypes obtained by two different crossing strategies. **c**, Representative H&E images of E8.5 embryos of the indicated genotypes. Scale bar=200μm. **d**, Genotyping PCR of embryos shown in (**b**) DNA from the embryos were obtained by laser capture microdissection (LCM). Four pairs of primers were used to distinguish *Parp1 wildtype* (*Parp1* +) allele, *Parp1* knockout (*Parp1 ko*) allele, *Brca2 wildtype* (*Brca2* +) allele and *Brca2* knockout (*Brca2 ko*) alleles. Primers are listed in **Supplementary Note 1**. **e**, Table showing numbers of E10.5 embryos with indicated genotypes obtained by the indicated crossing strategy.

a

E8.5									
$Parp1^{ko/+}$; $Brca2^{ko/+}$ x $Parp1^{ko/+}$; $Brca2^{ko/+}$	$Parp1^{+/+}$; $Brca2^{+/+}$	$Parp1^{+/+}$; $Brca2^{ko/+}$	$Parp1^{+/+}$; $Brca2^{ko/ko}$	$Parp1^{ko/+}$; $Brca2^{+/+}$	$Parp1^{ko/+}$; $Brca2^{ko/+}$	$Parp1^{ko/+}$; $Brca2^{ko/ko}$	$Parp1^{ko/ko}$; $Brca2^{+/+}$	$Parp1^{ko/ko}$; $Brca2^{ko/+}$	$Parp1^{ko/ko}$; $Brca2^{ko/ko}$	Total
Phenotype	normal	normal	abnormal	normal	normal	partial rescue	normal	normal	N/A	
Observed	4	7	5	4	13	3	1	7	0	44
Expected (ratio)	2.75 (1/16)	5.5 (1/8)	2.75 (1/16)	5.5 (1/8)	11 (1/4)	5.5 (1/8)	2.75 (1/16)	5.5 (1/8)	2.75 (1/16)	

b

E8.5							
$Parp1^{ko/+}$; $Brca2^{ko/+}$ x $Parp1^{ko/ko}$; $Brca2^{ko/+}$	$Parp1^{ko/+}$; $Brca2^{+/+}$	$Parp1^{ko/+}$; $Brca2^{ko/+}$	$Parp1^{ko/+}$; $Brca2^{ko/ko}$	$Parp1^{ko/ko}$; $Brca2^{+/+}$	$Parp1^{ko/ko}$; $Brca2^{ko/+}$	$Parp1^{ko/ko}$; $Brca2^{ko/ko}$	Total
Phenotype	normal	normal	partial rescue	normal	normal	N/A	
Observed	10	21	8	5	20	0	64
Expected (ratio)	8 (1/8)	16 (1/4)	8 (1/8)	8 (1/8)	16 (1/4)	8 (1/8)	
P value (χ^2-test)	0.0323*						

c

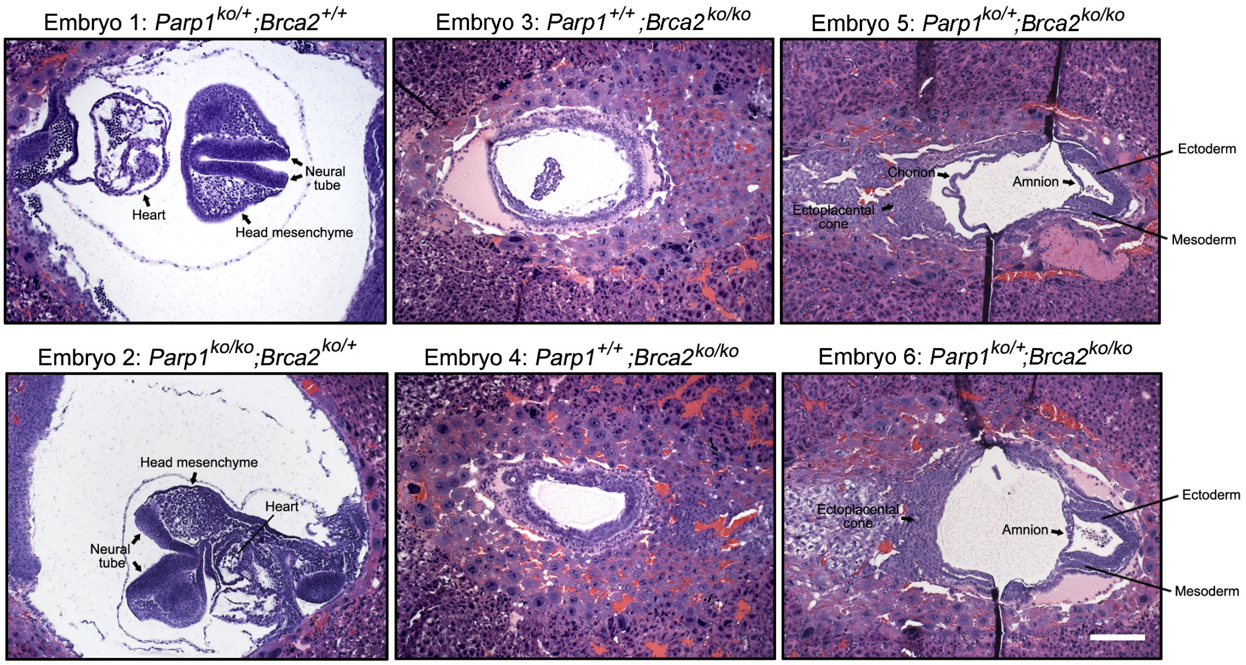

d

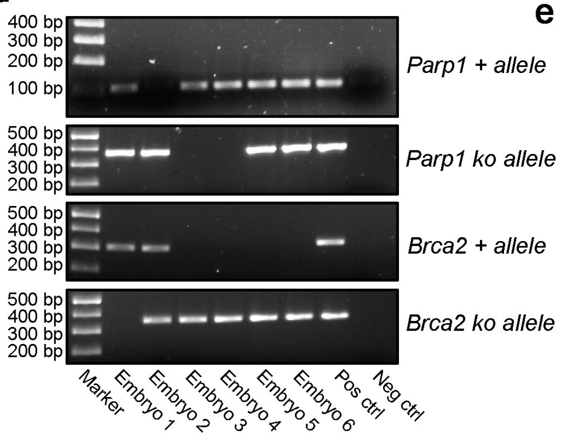

e

E10.5				
$Parp1^{ko/ko}$; $Brca2^{ko/+}$ x $Parp1^{+/+}$; $Brca2^{ko/+}$	$Parp1^{ko/+}$; $Brca2^{+/+}$	$Parp1^{ko/+}$; $Brca2^{ko/+}$	$Parp1^{ko/+}$; $Brca2^{ko/ko}$	Total
Phenotype	normal	normal	N/A	
Observed	4	15	0	19
Expected (ratio)	4.75 (1/4)	9.5 (1/2)	4.75 (1/4)	
P value (χ^2-test)	0.0178*			

Supplementary Figure 3

Supplementary Figure 4│*Brca2*$^{ko/ko}$ mESC rescued by PARP1 deficiency/PARP inhibition did not form RAD51 foci in response to IR and had increased genomic instability. **a**, Quantification of RAD51 foci immunofluorescence as shown in Fig.**2a**. All the cells were treated with 10 Gy, IR for 5 hours. Actual numbers of cells being counted are shown above each column. **b**, Western blot showing total RAD51 protein level in the indicated cells. **c**, Metaphase spread showing the karyotype of indicated cells. Red arrows point to chromosomal aberrations. **d**, Quantification of breaks/gaps. *$P<0.05$, **$P<0.01$ (*t*-test). **e**. Scattered plot showing increased sister chromatid exchange in PL2F7 cells under 1 μM, 16 hour-olaparib treatment. ****$P<0.0001$ (Mann-Whitney test).

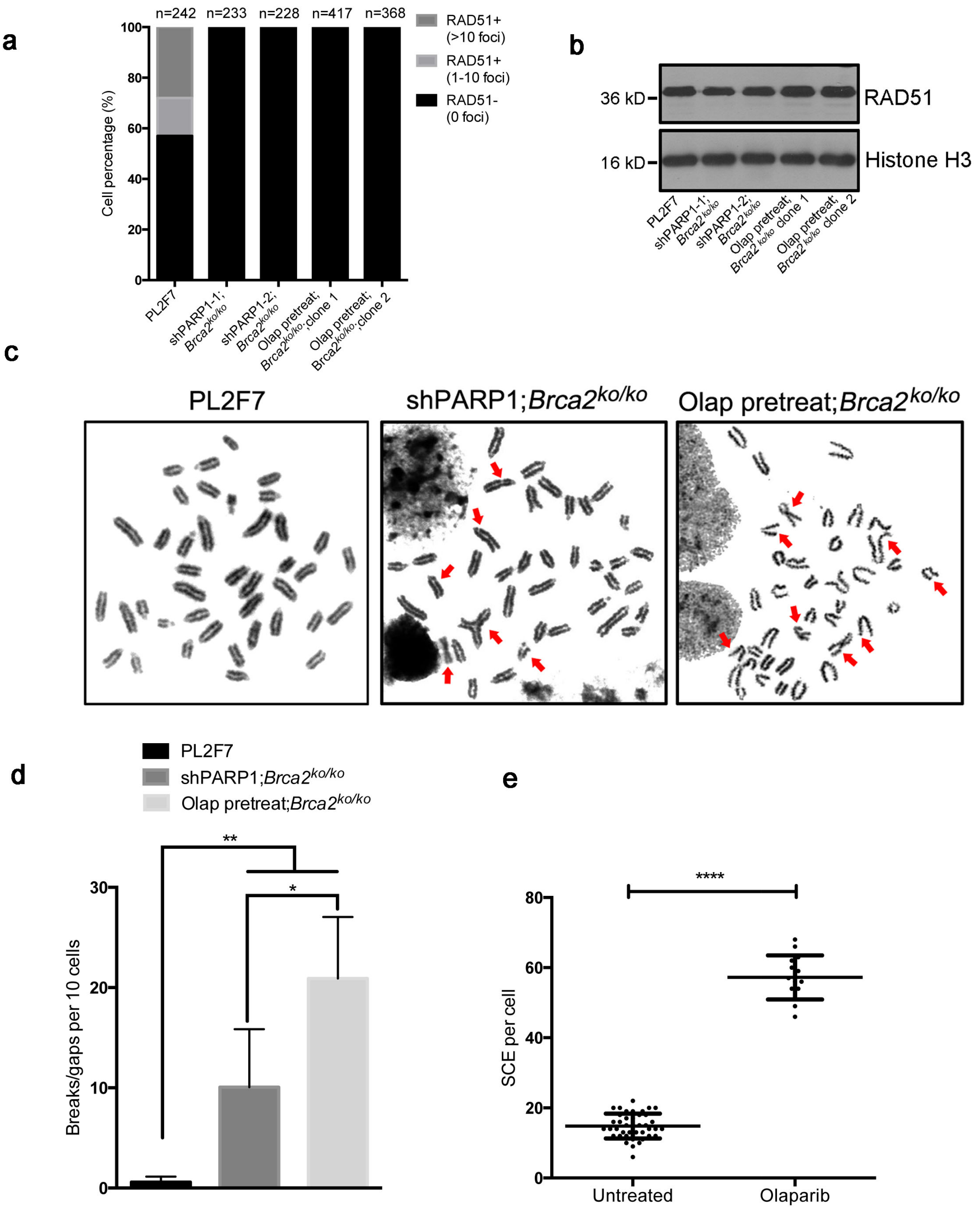

Supplementary Figure 4

Supplementary Figure 5 | Interaction between MRE11 and PARP1. **a**, Schematic representation of mouse PARP1 protein showing various functional/structural domains. FL, full length. **b**, Western blot of immunoprecipitation in HEK293T cells showing interaction between different PARP1 fragments and MRE11. Arrows point to the bands with correct size. **c**, Western blot of immunoprecipitation in HEK293T cells showing interaction between PARP1 N-terminal fragment and MRE11 with or without olaparib (10 μM, 3 hours). **d**, Western blot of immunoprecipitation in HEK293T cells showing interaction between PARP1 and MRE11 with or without DNase I.

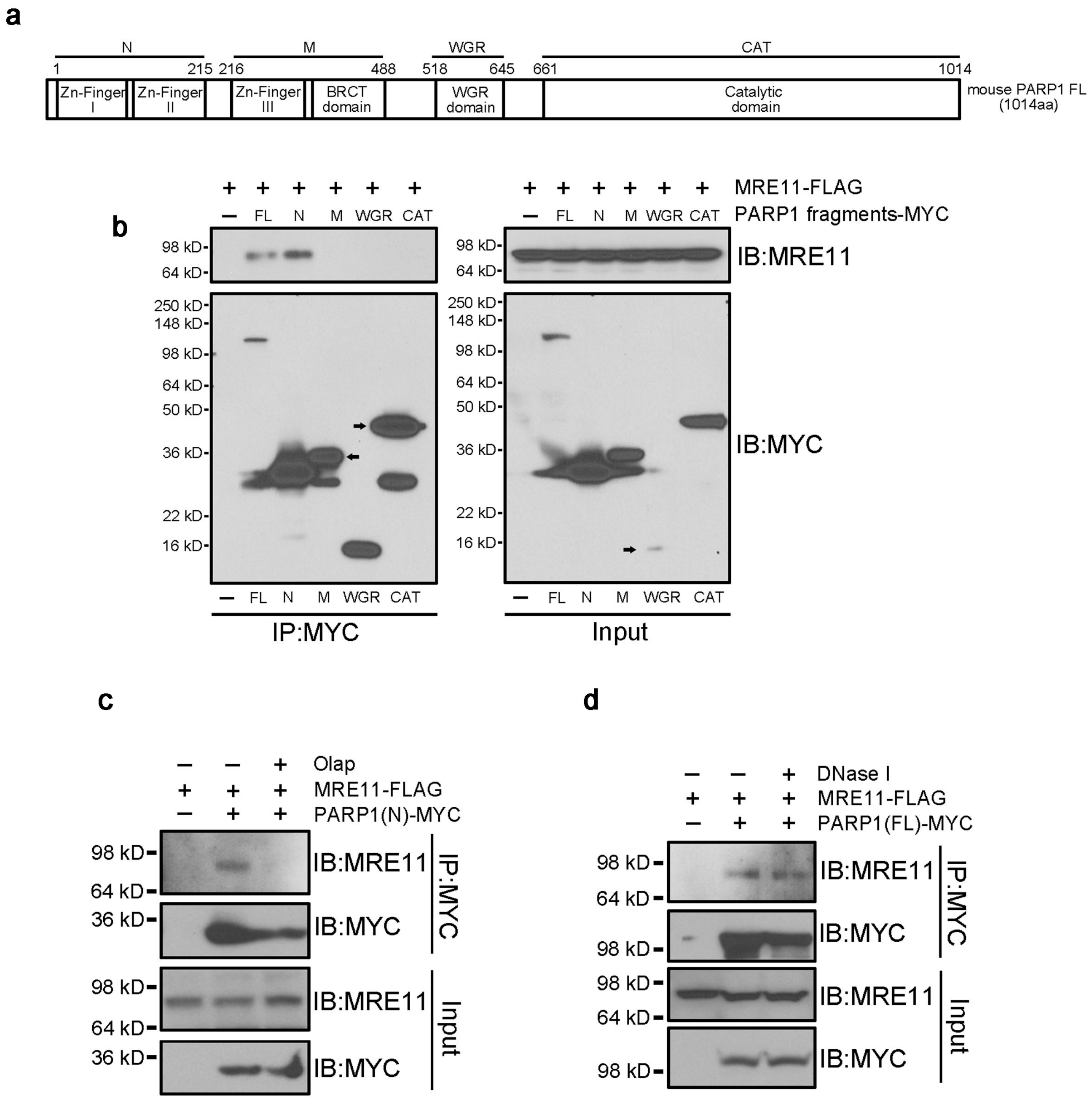

Supplementary Figure 5

Supplementary Figure 6│Transient inhibition of MRE11-mediated fork degradation by PARP inhibition may contribute to rescue *Brca2*$^{ko/ko}$ mESC. a, Scattered plot showing DNA fiber analysis of *BRCA2* mutant cells (Y3308X) treated with mirin or olaparib. ****P<0.0001 (Mann-Whitney test). **b**, Western blot showing PARP1 level in *wildtype* and *Parp1*$^{ko/ko}$ MEF cells. **c,** Western blot showing PARP1 level in *Brca2*$^{ko/ko}$ mESC rescued by PARP1 knockdown. **d**, Scattered plot showing DNA fiber analysis of *Brca2*$^{ko/ko}$ mESC rescued by olaparib pretreatment. ****P<0.0001 (Mann-Whitney test). **e**, Western blot of iPOND samples from PL2F7 cells and PARP1 stable knockdown clone with or without HU (4 mM, 4 hours) probed by MRE11. **f** and **h**, Representative images and quantification of RAD51 foci immunofluorescence after IR (10 Gy, 5 hours) in *Brca2*$^{ko/ko}$ mESC rescued by mirin or MRE11 knockdown. **g**, Representative images of DNA fibers as quantified in (**d**).

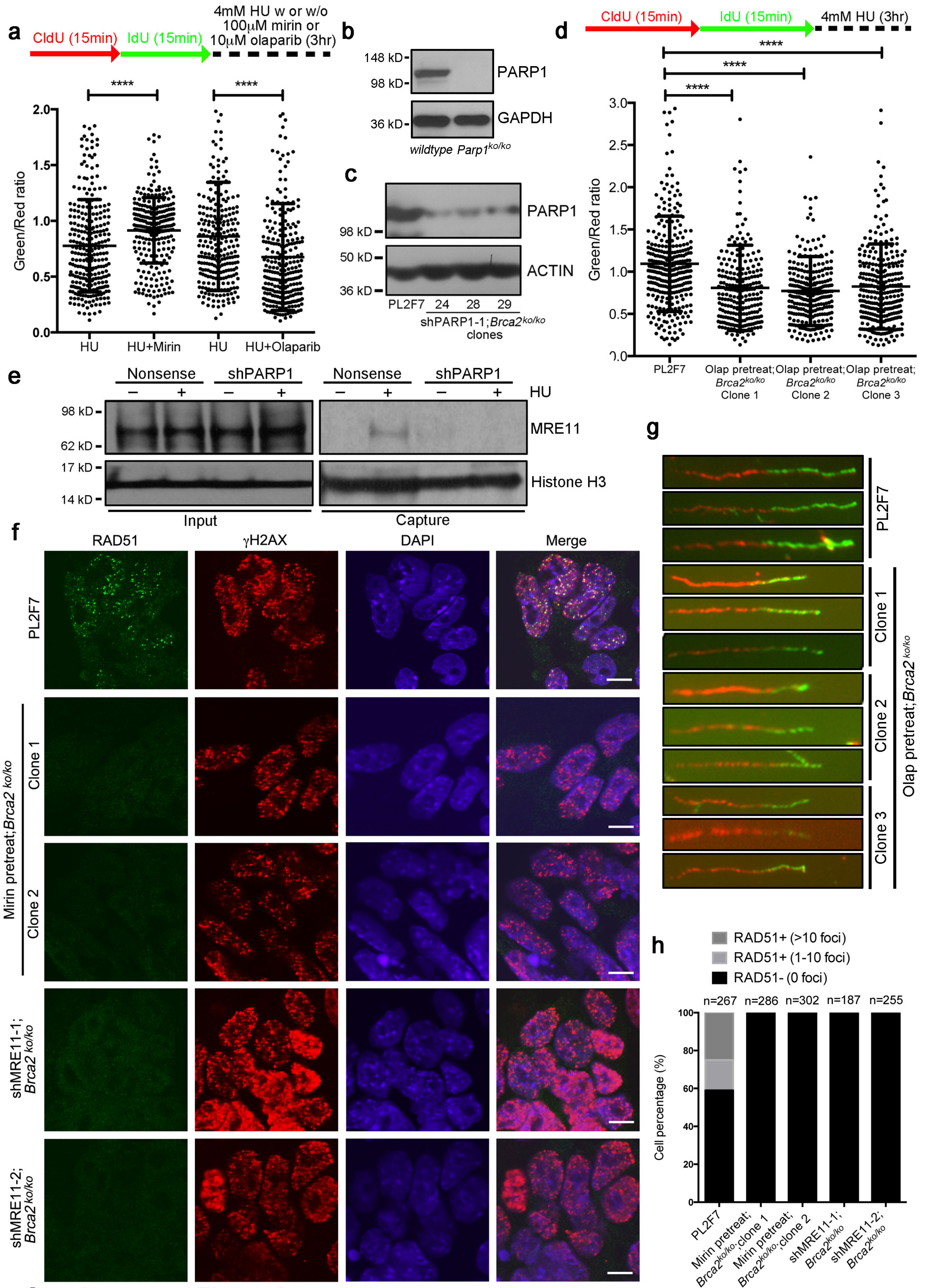

Supplementary Figure 6

Supplementary Figure 7│ H&E staining of tumors from *Parp1$^{ko/+}$;K14-Cre;Brca2$^{cko/cko}$* and *Parp1$^{+/+}$;K14-Cre;Brca2$^{cko/cko}$* mice. Scale bars=100 μm.

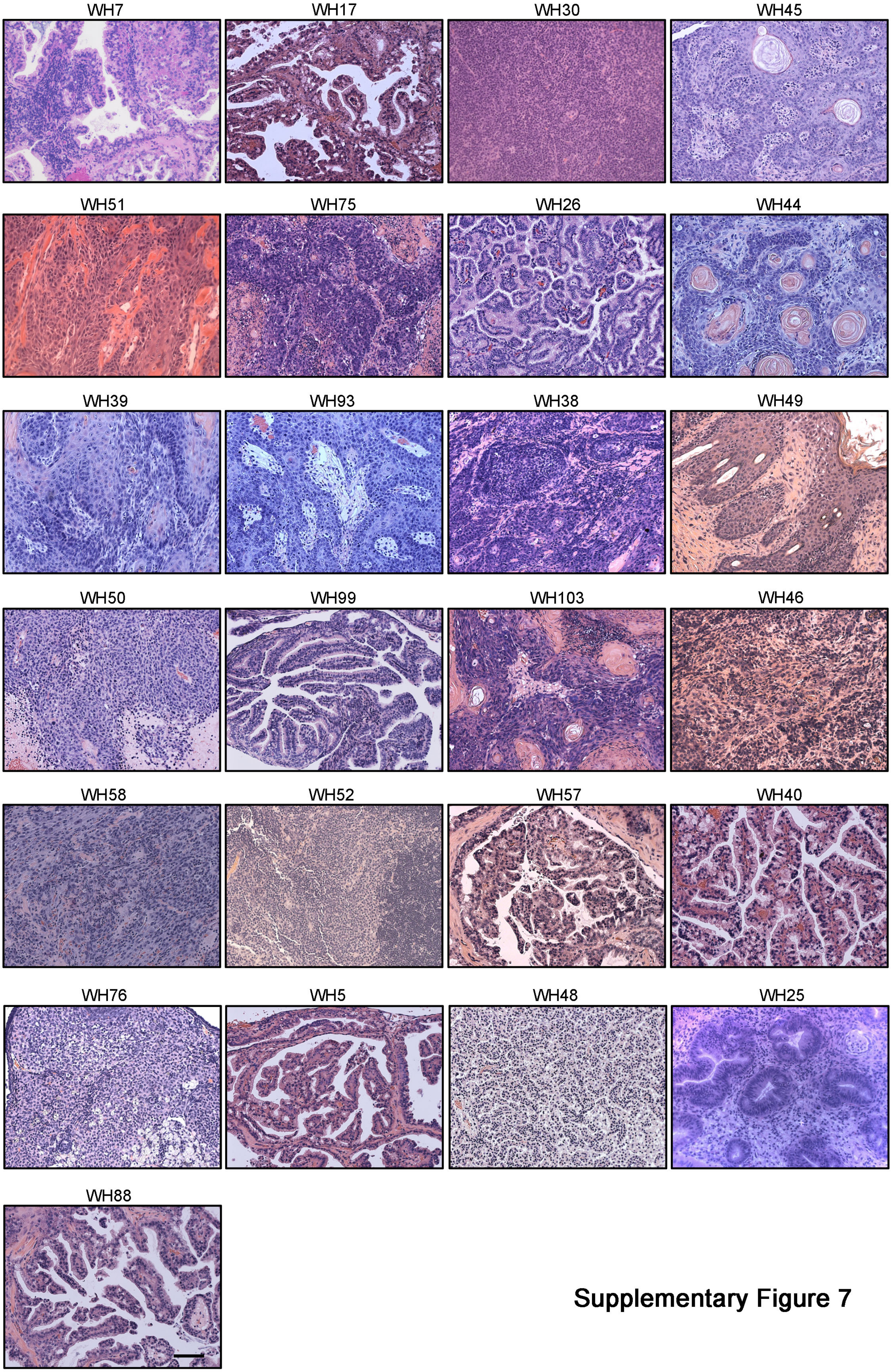

Supplementary Figure 7

Supplementary Figure 8 | Uncropped image of the scanned blots

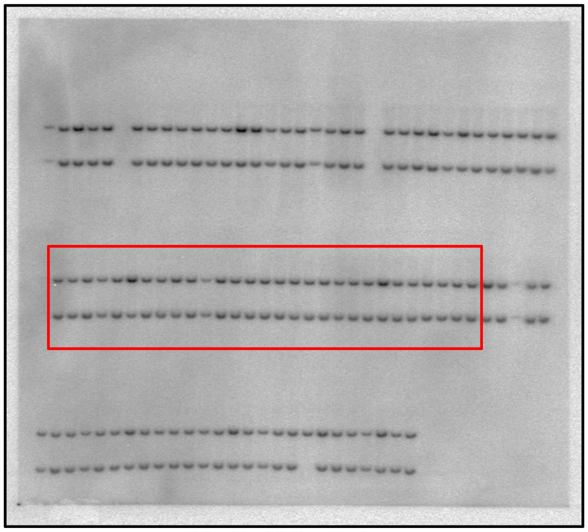

Fig 1b. Untreated

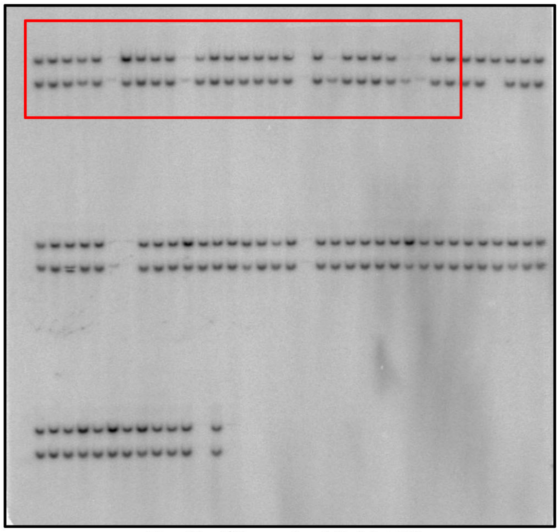

Fig 1b. Olaparib, 10nM, 72hr

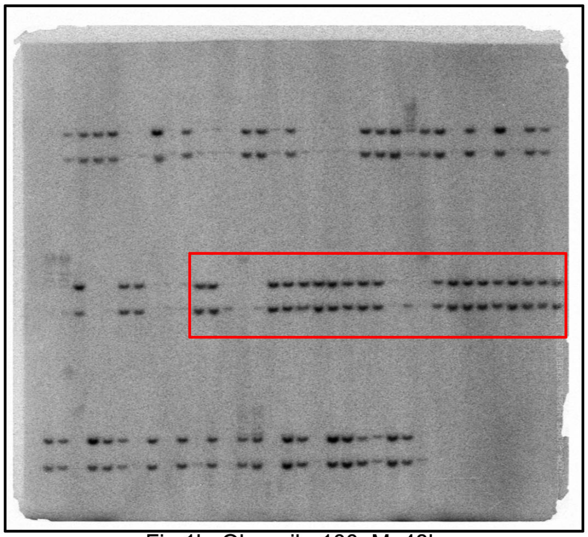

Fig 1b. Olaparib, 100nM, 48hr

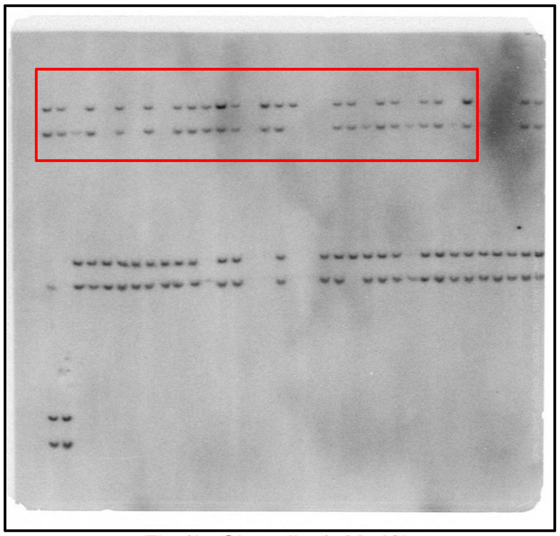

Fig 1b. Olaparib, 1μM, 48hr

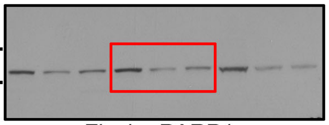

Fig 1c. PARP1

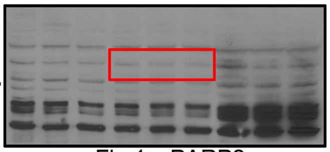

Fig 1c. PARP2

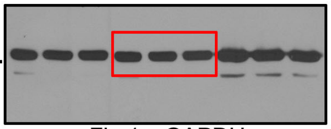

Fig 1c. GAPDH

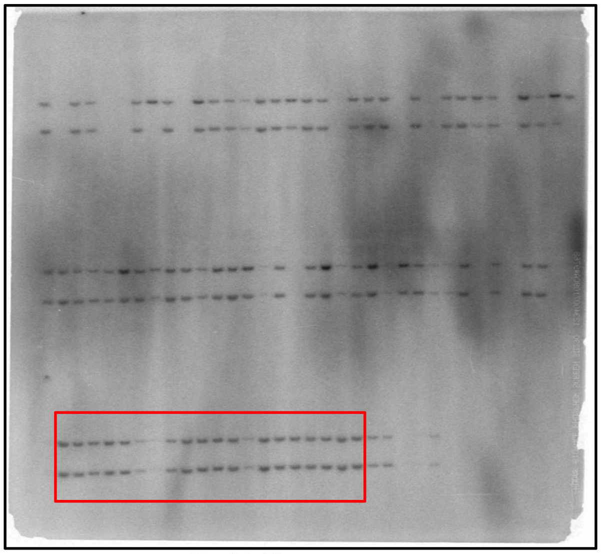

Fig 1d. Nonsense

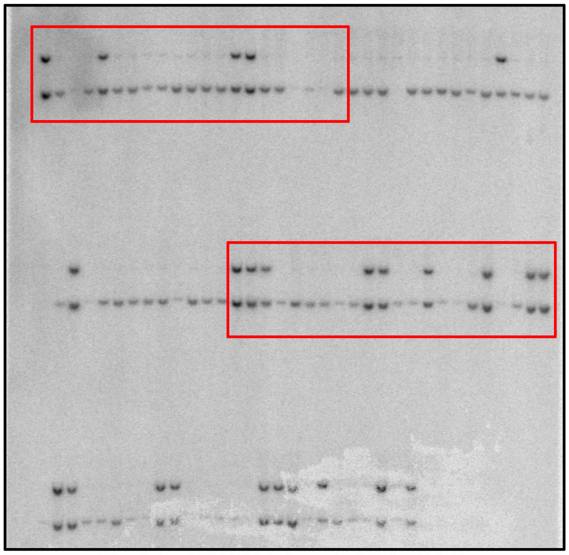

Fig 1d. shPARP1-1 and shPARP1-2

Supplementary Figure 8 part 1

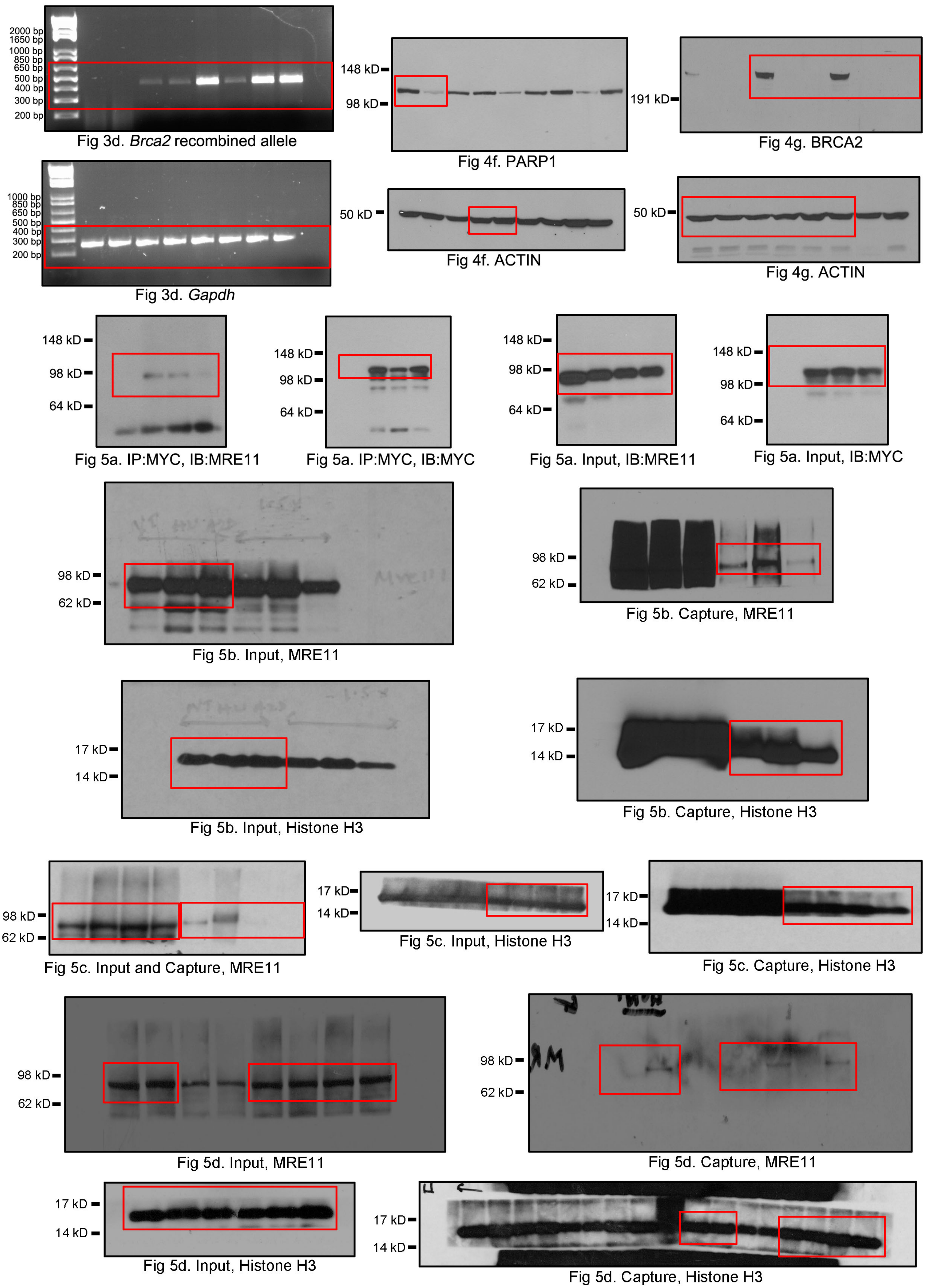

Supplementary Figure 8 part 2

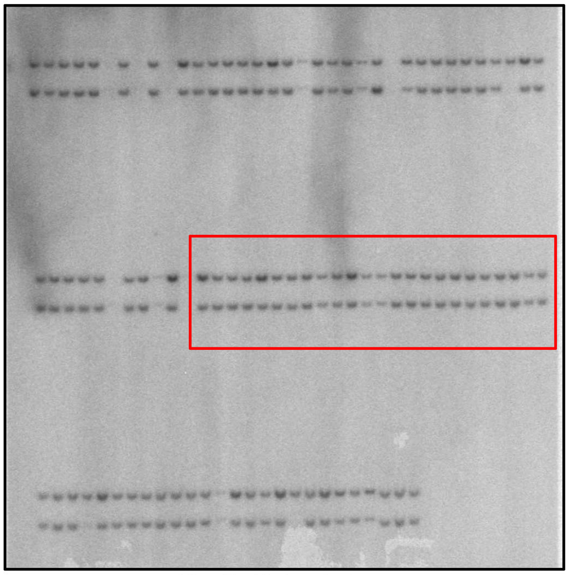

Fig. 5e Untreated

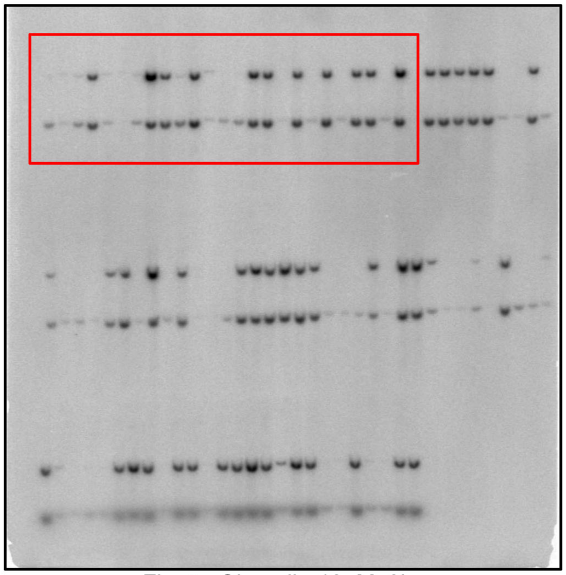

Fig. 5e Olaparib, 10μM, 3hr

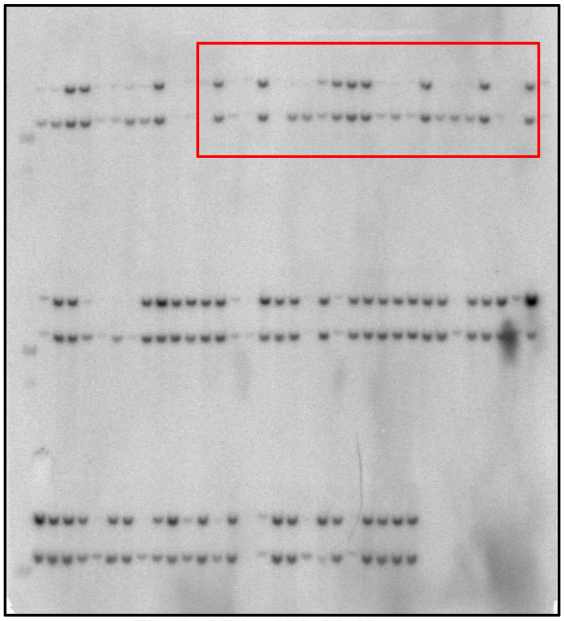

Fig. 5e Mirin, 100μM, 3hr

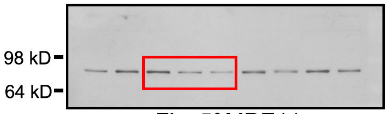

Fig. 5f MRE11

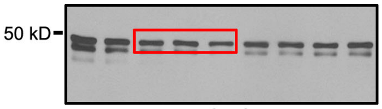

Fig. 5f ACTIN

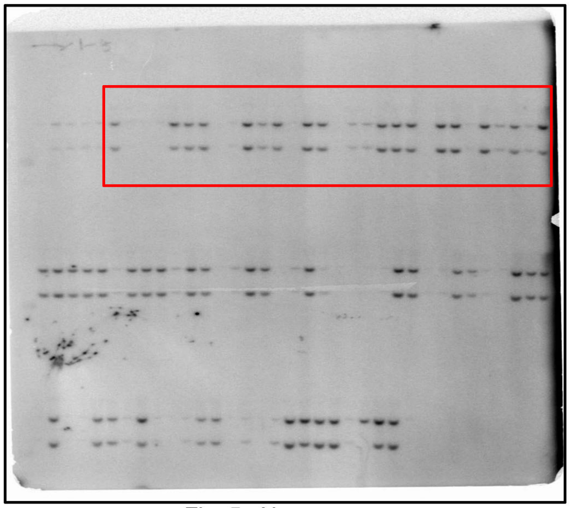

Fig. 5g Nonsense

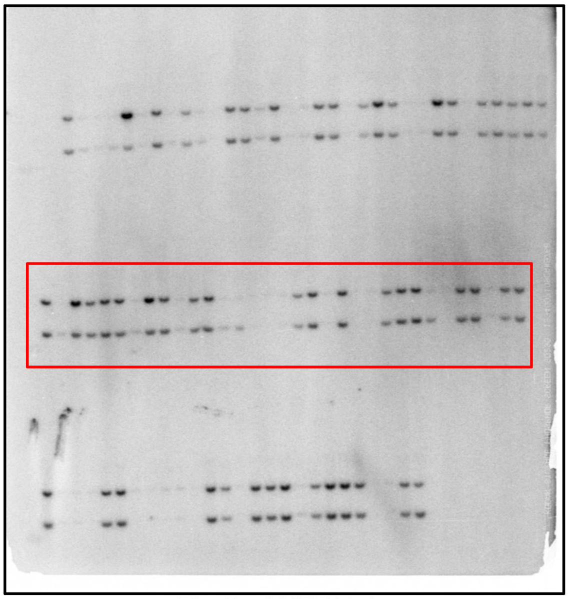

Fig. 5g shMRE11-1

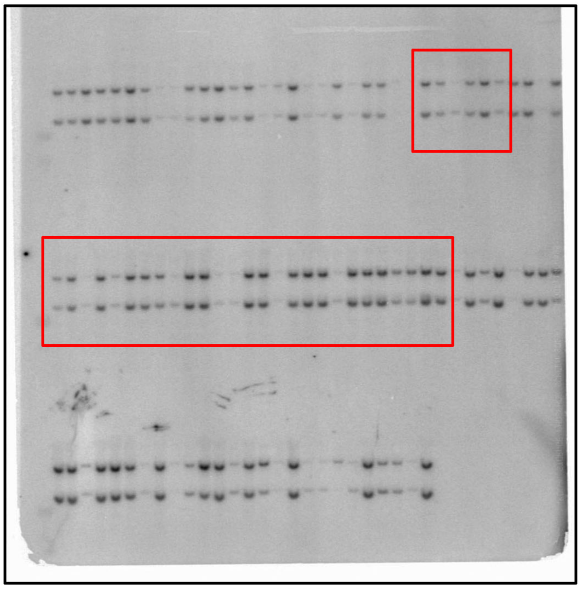

Fig. 5g shMRE11-2

Supplementary Figure 8 part 3

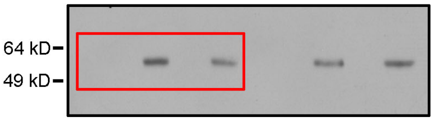

Supplementary Fig.1a
10nM olap, pS317 CHK1

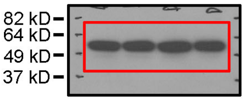

Supplementary Fig.1a
10nM olap, total CHK1

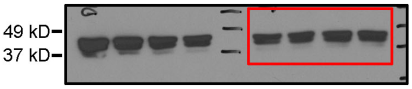

Supplementary Fig. 1a
10nM olap, ACTIN

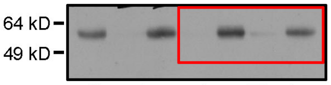

Supplementary Fig.1a
100nM olap, pS317 CHK1

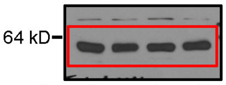

Supplementary Fig.1a
100nM olap, total CHK1

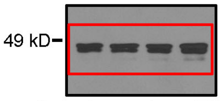

Supplementary Fig.1a
100nM olap, ACTIN

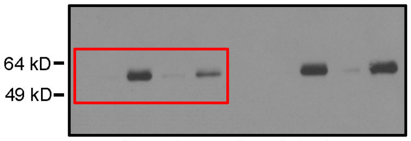

Supplementary Fig.1a
1μM olap, pS317 CHK1

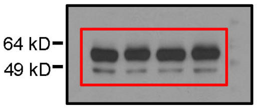

Supplementary Fig.1a
1μM olap, total CHK1

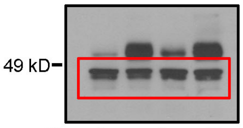

Supplementary Fig.1a
1μM olap, ACTIN

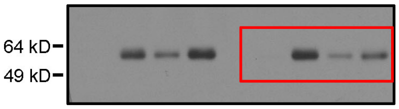

Supplementary Fig.1a
10μM olap, pS317 CHK1

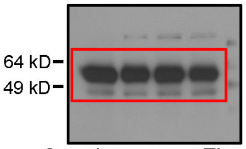

Supplementary Fig.1a
10μM olap, total CHK1

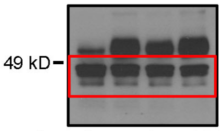

Supplementary Fig.1a
10μM olap, ACTIN

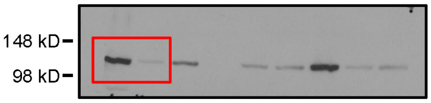

Supplementary Fig.1e
PARP1

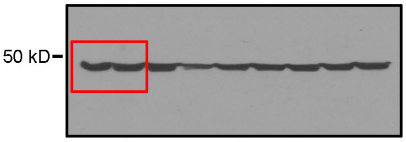

Supplementary Fig.1e
ACTIN

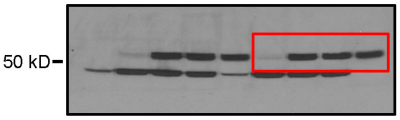

Supplementary Fig.1h
pS15 p53

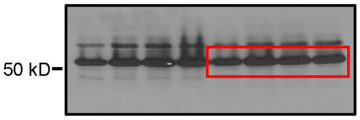

Supplementary Fig.1h
Total p53

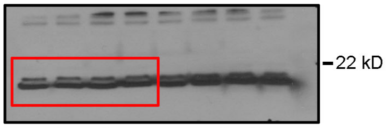

Supplementary Fig.1h
p19

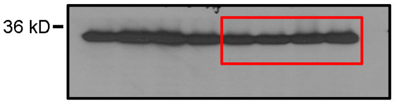

Supplementary Fig.1h
PCNA

Supplementary Figure 8 part 4

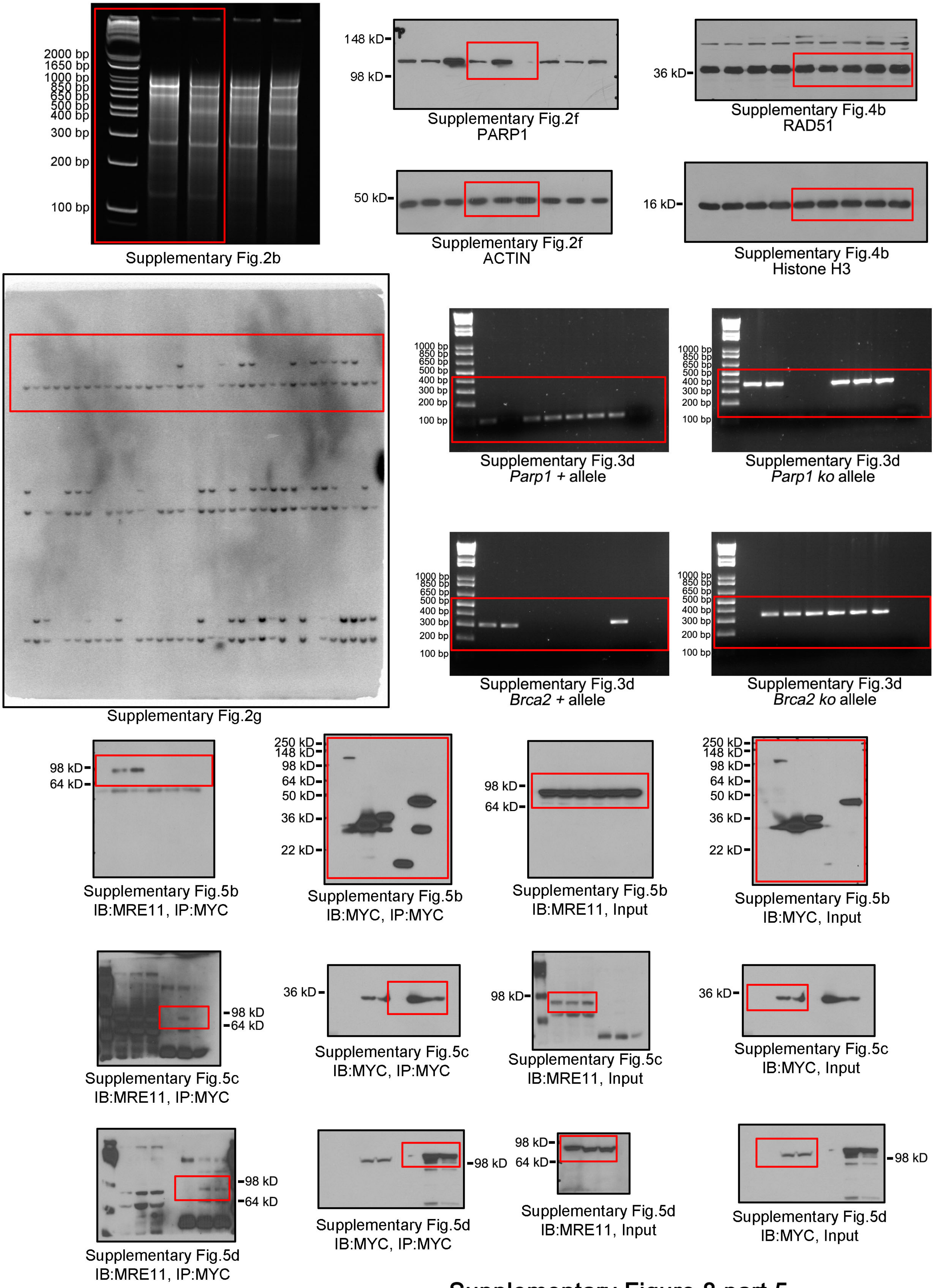

Supplementary Figure 8 part 5

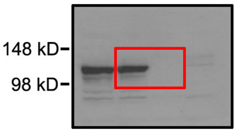

Supplementary Fig.6b
PARP1

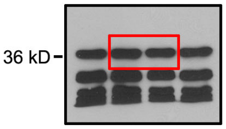

Supplementary Fig.6b
GAPDH

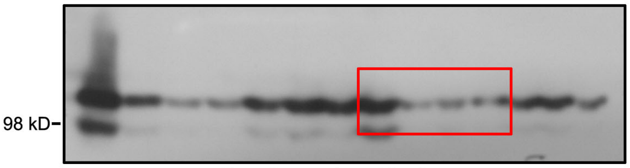

Supplementary Fig.6c
PARP1

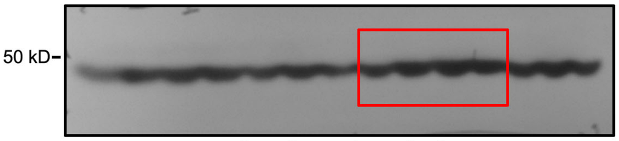

Supplementary Fig.6c
ACTIN

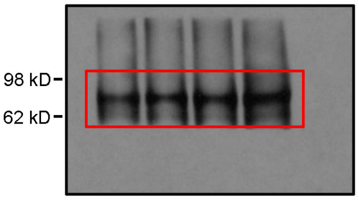

Supplementary Fig 6e.
Input, MRE11

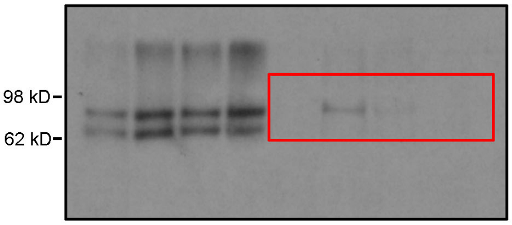

Supplementary Fig. 6e.
Capture, MRE11

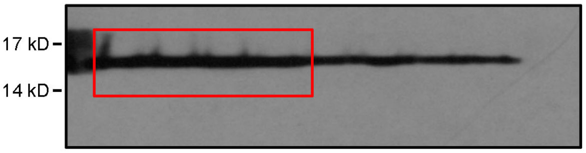

Supplementary Fig 6e.
Input, Histone H3

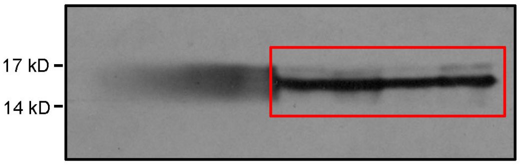

Supplementary Fig 6e.
Capture, Histone H3

Supplementary Table 1 Table showing the age, sex, tumor type and genotype of mice that developed tumor.

Mice #	Age of tumor onset (days)	Sex	Tumor type	Genotypes
WH7	611	Male	Harderian gland adenoma	*$Parp1^{ko/+}$;K14-Cre;$Brca2^{cko/cko}$*
WH17	428	Male	Harderian gland adenoma	
WH30	262	Female	Skin tumor	
WH45	414	Male	Zymbal's gland carcinoma	
WH51	379	Male	Skin tumor	
WH75	424	Male	Oral cavity squamous cell carcinoma	
WH26	535	Male	Lung alveolar carcinoma	
WH44	527	Male	Skin squamous cell carcinoma	
WH39	558	Female	Pinna squamous cell carcinoma	
WH93	450	Male	Pinna squamous cell carcinoma	
WH38	568	Female	Skin squamous cell carcinoma and harderian gland adenoma	
WH49	547	Male	Pinna squamous cell carcinoma	
WH50	561	Male	Skin squamous cell carcinoma	
WH99	395	Female	Lung alveolar adenoma	
WH103	444	Male	Zymbal's gland carcinoma	
WH46	582	Male	Salivary gland myoepithelioma	
WH58	586	Male	Skin squamous cell carcinoma	
WH52	530	Female	Skin squamous cell papilloma	
WH57	530	Male	Harderian gland adenoma	
WH40	568	Male	Harderian gland adenoma	
WH76	587	Female	Skin squamous cell carcinoma	
WH5	734	Male	Harderian gland adenoma	
WH48	645	Male	Lung alveolar adenoma	
WH25	465	Male	Stomach glandular adenoma	*$Parp1^{+/+}$;K14-Cre;$Brca2^{cko/cko}$*
WH88	465	Male	Harderian gland adenoma	

Supplementary Note 1 | List of oligonucleotides

Oligo-1. shRNA-1 against mouse *Parp1* mRNA (sense, targeted sequences are underlined):
5'GATCCCCGGATGAAATTAACTCTGAATTCAAGAGATTCAGAGTTAATTTCATCCTTTTTA

Oligo-2. shRNA-1 against mouse *Parp1* mRNA (antisense, targeted sequences are underlined):
5'AGCTTAAAAAGGATGAAATTAACTCTGAATCTCTTGAATTCAGAGTTAATTTCATCCGGG

Oligo-3. shRNA-2 against mouse *Parp1* mRNA (sense, targeted sequences are underlined):
5'GATCCCCGGACAAGGATAGTAGTAAGTTCAAGAGACTTACTACTATCCTTGTCCTTTTTA

Oligo-4. shRNA-2 against mouse *Parp1* mRNA (antisense, targeted sequences are underlined):
5'AGCTTAAAAAGGACAAGGATAGTAGTAAGTCTCTTGAACTTACTACTATCCTTGTCCGGG

Oligo-5. Control nonsense shRNA (sense, targeted sequences are underlined):
5'GATCCCCACTACCGTTGTTATAGGTGTTCAAGAGACACCTATAACAACGGTAGTTTTTA

Oligo-6. Control nonsense shRNA (antisense, targeted sequences are underlined):
5'AGCTTAAAAAACTACCGTTGTTATAGGTGTCTCTTGAACACCTATAACAACGGTAGTGGG

Oligo-7. sgRNA targeting mouse *Parp1* exon2 (upstream, top strand, targeted sequences are underlined):
5' CACCGCTCAACATCAGGCTGCCGGA

Oligo-8. sgRNA targeting mouse *Parp1* exon2 (upstream, bottom strand, targeted sequences are underlined):
5' AAACTCCGGCAGCCTGATGTTGAGC

Oligo-9. sgRNA targeting mouse *Parp1* exon2 (downstream, top strand, targeted sequences are underlined):
5' CACCGATGGCTTCTCTGAGCTGCGC

Oligo-10. sgRNA targeting mouse *Parp1* exon2 (downstream, bottom strand, targeted sequences are underlined):
5' AAACGCGCAGCTCAGAGAAGCCATC

Supplementary Note 1

Oligo-11. PCR primer for mouse *Parp1* exon2 sgRNA SURVEYOR nuclease assay (forward)
5’ GGCCATCCAGACCCTTGAGTTCAAGTG

Oligo-12. PCR primer for mouse *Parp1* exon2 sgRNA SURVEYOR nuclease assay (reverse)
5’ ACTAGGTTCTATCAGACGCACTGGTGG

(Note: **Oligo-11** and **Oligo-12** generate 931bp PCR product)

Oligo-13. PCR primer for mouse *Parp1* exon2 sgRNA mESC sequencing (forward)
5’ ATACCCAGGATGAGAAGCCAGAAGC

Oligo-14. PCR primer for mouse *Parp1* exon2 sgRNA mESC sequencing (reverse)
5’ TAAAGTCTTCCTTTCCATCATCTGG

(Note: **Oligo-13** and **Oligo-14** generate 456bp PCR product)

Oligo-15. PCR primer for genotyping *Brca2 ko* allele in $Brca2^{ko/+}$ mice (forward)
5’ GTGAATCTTTGTCAGCAGTTCCC

Oligo-16. PCR primer for genotyping *Brca2 ko* allele in $Brca2^{ko/+}$ mice (reverse)
5’ CCCACTAGCTGTATGAAAAC

(Note: **Oligo-15** and **Oligo-16** generate approximately 340bp PCR product)

Oligo-17. PCR primer for genotyping *Brca2* + allele in $Brca2^{ko/+}$ mice (forward)
5’ GCAAAAGTAGGACCAAGAGG

Oligo-18. PCR primer for genotyping *Brca2* + allele in $Brca2^{ko/+}$ mice (reverse)
5’ TCACCTTTATGAATATAAACTG

(Note: **Oligo-17** and **Oligo-18** generate approximately 300bp PCR product)

Oligo-19. PCR primer for genotyping $Brca2^{cko/cko}$ mice (forward)
5’ CTCATCATTTGTTGCCTCACTTC

Oligo-20. PCR primer for genotyping $Brca2^{cko/cko}$ mice (reverse)
5’ TGTTGGATACAAGGCATGTAC

Oligo-21. PCR primers for detecting mouse genomic *Gapdh* (forward)

5’ ACCAGGGCTGCCATTTGCAGTGGC

Oligo-22. PCR primers for detecting mouse genomic *Gapdh* (reverse)

Supplementary Note 1

5' CTTCTCCATGGTGGTGAAGACACC

(Note: **Oligo-19** and **Oligo-20** generate 449bp PCR product for *wildtype* allele and a slightly larger PCR product for *cko* allele because of *loxP* site integration. **Oligo-21** and **Oligo-22** amplify genomic *Gapdh* as control, product size is 267bp. These primers are also used for quantitative PCR to detect *Brca2* genomic deletion in B cells)

Oligo-23. PCR primer for genotyping in $Parp1^{ko/+}$ mice (common forward)
5' CATGTTCGATGGGAAAGTCCC

Oligo-24. PCR primer for genotyping *Parp1 ko* allele in $Parp1^{ko/+}$ mice (reverse)
5' AGGTGAGATGACAGGAGATC

Oligo-25. PCR primer for genotyping *Parp1* + allele in $Parp1^{ko/+}$ mice (reverse)
5' CCAGCGCAGCTCAGAGAAGCCA

(Note: **Oligo-23** and **Oligo-24** generate 350bp PCR product for *Parp1 ko* allele, **Oligo-23** and **Oligo-25** generate 112bp PCR product for *Parp1* + allele)

(Note: **Oligo-17, -18, -23, -24, -25** are also the primers used for embryo genotyping and LCM sample genotyping)

Oligo-26. PCR primer for genotyping *CD19-Cre* mice (*Cre* forward)
5' TGGTTTCCCGCAGAACCTGAAG

Oligo-27. PCR primer for genotyping *CD19-Cre* mice (*Cre* reverse)
5' GAGCCTGTTTTGCACGTTCACC

Oligo-28. PCR primer for genotyping *CD19-Cre* mice (*CD19* forward)
5' ACTCACCACTATCCTCCACGTT

Oligo-29. PCR primer for genotyping *CD19-Cre* mice (*CD19* reverse)
5' CAATGTTGTGCTGCCATGCCT

(Note: **Oligo-26** and **Oligo-27** generate approximately 200bp PCR product for *Cre*, **Oligo-28** and **Oligo-29** generate 266bp PCR product for *CD19*. Wildtype mice only have *CD19* band, heterozygous mice have both *CD19* and *Cre* bands, *CD19-Cre* homozygous mice have only *Cre* band)

Oligo-30. PCR primer for genotyping *K14-Cre* mice (forward)
5' CCATCTGCCACCAGCCAG

Oligo-31. PCR primer for genotyping *K14-Cre* mice (reverse)
5' TCGCCATCTTCCAGCAGG

Supplementary Note 1

(Note: **Oligo-30** and **Oligo-31** generate 281bp PCR product for *K14-Cre* transgene positive mice)

Oligo-32. shRNA-1 against mouse *Mre11* mRNA (sense, targeted sequences are underlined):

5'GATCCCCTCCGACTACGGGTGGACTATTCAAGAGATAGTCCACCCGTAGTCGGATTTTTA

Oligo-33. shRNA-1 against mouse *Mre11* mRNA (antisense, targeted sequences are underlined):

5'AGCTTAAAAATCCGACTACGGGTGGACTATCTCTTGAATAGTCCACCCGTAGTCGGAGGG

Oligo-34. shRNA-2 against mouse *Mre11* mRNA (sense, targeted sequences are underlined):

5'GATCCCCGTAGGCTTGCTGCGCATTATTCAAGAGATAATGCGCAGCAAGCCTACTTTTTA

Oligo-35. shRNA-2 against mouse *Mre11* mRNA (antisense, targeted sequences are underlined):

5'AGCTTAAAAAGTAGGCTTGCTGCGCATTATCTCTTGAATAATGCGCAGCAAGCCTACGGG

Oligo-36: PCR primer for genotyping *Brca2* recombined allele in hematopoietic progenitor cell clones (forward)

5' GGCTGTCTTAGAACTTAGGCT

Oligo-37: PCR primer for genotyping *Brca2* recombined allele in hematopoietic progenitor cell clones (reverse)

5' TGTTGGATACAAGGCATGTAC

(Note: **Oligo-36** and **Oligo-37** amplify approx. 450bp PCR product if *Brca2 cko* allele has undergone recombination. **Oligo-21** and **Oligo-22** are used to detect genomic *Gapdh* as control)

Oligo-38: PCR primes for generating mouse *Parp1* full-length (FL) cDNA from MGC mouse *Parp1* cDNA clone (forward).

5' CCCAAGCTTGGGATGGCGGAGGCCTCGGAGAGG

Supplementary Note 1

Oligo-39: PCR primes for generating mouse *Parp1* full-length (FL) cDNA from MGC mouse *Parp1* cDNA clone (reverse).

5'CCGCTCGAGCGGTCACAGATCCTCTTCTGAGATGAGTTTTTGTTCCCACAGG GATGTC

Oligo-40: PCR primes for generating mouse *Parp1* N-fragment cDNA from MGC mouse *Parp1* cDNA clone (forward).

5' CCCAAGCTTGGGATGGCGGAGGCCTCGGAGAGG

Oligo-41: PCR primes for generating mouse *Parp1* N-fragment cDNA from MGC mouse *Parp1* cDNA clone (reverse).

5'CCGCTCGAGCGGTCACAGATCCTCTTCTGAGATGAGTTTTTGTTCTCCATCC ACCTCGTC

Oligo-42: PCR primes for generating mouse *Parp1* M-fragment cDNA from MGC mouse *Parp1* cDNA clone (forward).

5' CCCAAGCTTGGGATGACAGATGAAGTGGCCAAAAAG

Oligo-43: PCR primes for generating mouse *Parp1* M-fragment cDNA from MGC mouse *Parp1* cDNA clone (reverse).

5'CCGCTCGAGCGGTCACAGATCCTCTTCTGAGATGAGTTTTTGTTCTGCCTTC ACCTCAGC

Oligo-44: PCR primes for generating mouse *Parp1* WGR-fragment cDNA from MGC mouse *Parp1* cDNA clone (forward).

5' CCCAAGCTTGGGATGAAATCTGAAAAGAGGATG

Oligo-45: PCR primes for generating mouse *Parp1* WGR-fragment cDNA from MGC mouse *Parp1* cDNA clone (reverse).

5'CCGCTCGAGCGGTCACAGATCCTCTTCTGAGATGAGTTTTTGTTCATAGTCA ATCTCCAG

Oligo-46: PCR primes for generating mouse *Parp1* CAT-fragment cDNA from MGC mouse *Parp1* cDNA clone (forward).

5' CCCAAGCTTGGGATGACCAAGTCGAAGCTGCCG

Supplementary Note 1

Oligo-47: PCR primes for generating mouse *Parp1* CAT-fragment cDNA from MGC mouse *Parp1* cDNA clone (reverse).

5'CCGCTCGAGCGGTCACAGATCCTCTTCTGAGATGAGTTTTTGTTCCCACAGG GATGTC

(Note: **Oligo-38** to **Oligo-47** amplify mouse *Parp1* FL, N, M, WGR and CAT cDNA fragments, respectively. These primers add HindIII site to 5' end and a MYC tag with Xho I site to 3' end of all the fragments)
